# A complex reciprocal translocation is linked to reduced gamete viability in a loose-bunch grapevine somatic variant

**DOI:** 10.1186/s12870-026-08212-7

**Published:** 2026-01-27

**Authors:** Noelia Alañón-Sánchez, Yolanda Ferradás, Ilja Bezrukov, Detlef Weigel, Pablo Carbonell-Bejerano, Javier Ibáñez

**Affiliations:** 1 Instituto de Ciencias de la Vid y del Vino , ICVV; CSIC, Gobierno de La Rioja, Universidad de La Rioja, Logroño, 26007 Spain; 2 Facultade de Bioloxía, Universidade de Santiago de Compostela, Santiago de Compostela, 15872 Spain; 3 Max Planck Institute for Biology Tübingen, Tübingen, 72076 Germany; 4 Institute for Bioinformatics and Medical Informatics, University of Tübingen, Tübingen, 72076 Germany

**Keywords:** Bunch compactness, Clonal variation, Gamete viability, Genome structural variation, Grapevine, Long-read sequencing, Reciprocal translocation, Somatic genome rearrangement

## Abstract

**Background:**

Because grapevine (*Vitis vinifera* L.) cultivars are highly heterozygous, they must be clonally propagated to preserve their varietal attributes. Over extended cultivar propagation, somatic mutations arise and can generate new phenotypes useful for intra-varietal improvement. Somatic variants with looser bunches – associated with more uniform berry ripening and reduced bunch rot incidence – are particularly valuable in compact-bunch cultivars. To understand the basis of this trait, we combined phenotyping, genomics, and genetic analyses to study VP11, a loose-bunch somatic variant clone with reduced fruit set of the wine grape cultivar ‘Tempranillo Tinto’.

**Results:**

Pollen viability and the number of seeds per berry were reduced by ~ 50% in VP11 compared to a control clone of ‘Tempranillo Tinto’. Long-read whole-genome sequencing identified eleven large somatic structural variants (SVs) in VP11, including three heterozygous inter-chromosomal events. These consisted of one fixed reciprocal translocation (Tra1-3), with duplications spanning tens of kilobases at the translocation breakpoints, and two segmental duplications. All three SVs were molecularly validated, including the phasing and exchange of distal chromosome segments in Tra1-3. In VP11 self-cross progeny, pollen viability was significantly reduced among individuals carrying Tra1-3, and the two translocation chromosomes were always inherited together, indicating that gametes with an unbalanced chromosomal content are non-viable.

**Conclusions:**

This study identifies a heterozygous balanced reciprocal translocation linked to reduced gamete viability in a loose-bunch grapevine somatic variant with low fruit set. This finding suggests that genetic defects that reduce gamete viability might be selected in vegetatively propagated crops when decreased seed and fruit set confer agronomic benefits.

**Supplementary Information:**

The online version contains supplementary material available at 10.1186/s12870-026-08212-7.

## Background

Grapevine (*Vitis vinifera L.*) is one of the most important fruit crops, covering more than 7.3 million hectares worldwide [1]. Grapevine is the foremost basis of winemaking but also of table grapes, raisins and juices. Grapevine cultivars are vegetatively propagated to maintain their varietal attributes, and many cultivars have been cultivated for centuries. Throughout their history of cultivation, spontaneous somatic mutations have occurred, and these have been maintained in specific clonal lines during cycles of vegetative multiplication [2]. Some of the associated phenotypes improve traits of interest, offering opportunities for the improvement of traditional grape cultivars [3–5]. Unlike genetic crosses, which in highly heterozygous organisms like grapevine cultivars disrupt varietal identity, somatic variants allow variety improvement while preserving the agronomic and oenological characteristics of elite cultivars [5]. Somatic variants affecting quantitative traits, like bunch compactness, phenology or berry ripening parameters, can be fixed in new clones of the same cultivar for intra-varietal improvement [6]. Somatic variants that change qualitative traits such as berry color are considered new cultivars, such as the ‘Pinot Blanc’ or ‘Garnacha Blanca’ white varietals that derive from ‘Pinot Noir’ and ‘Garnacha Tinta’ red cultivars [4].

Bunch compactness, a trait that refers to the density and spatial arrangement of berries within a grape bunch [7], is agronomically important because it directly affects disease susceptibility and fruit quality. Compact bunches are more susceptible to pests and fungal diseases such as bunch rot caused by *Botrytis* *cinerea* [8, 9]. In addition, berry ripening within compact bunches is more uneven due to the establishment of micro-environment variations between covered inner and exposed outer berries, affecting the uniformity of berry composition at harvest and wine quality [10]. On the other hand, very loose bunches, in addition to resulting in lower yield, may be less favorable for mechanical harvesting or less preferred by consumers [11].

Variation in grapevine bunch compactness mainly depends on rachis architecture, berry size and berry number per bunch, which together determine how tightly the berries are packed within the bunch [7, 12, 13]. The density of berries in the grapevine bunch is critically determined by the number of flowers per inflorescence and the fraction of flowers that become fruits [14], and both are genotype-dependent [15]. Fruit set rate is in turn affected by climatic conditions, the nutritional status of the plant, as well as by developmental factors including gamete viability [16, 17]. In line with the different developmental processes involved, the genetic determination of bunch compactness is complex and governed by multiple loci [18–22].

The viability of male and female gametes in plants (pollen and ovule viability) plays a critical role in their reproductive success, influencing key outcomes such as fertilization efficiency, seed development, and fruit set. Reduced viability of male gametes is commonly associated with a concomitant decline in female fertility [23–25]. In various grapevine cultivars and clonal variants, limited pollen viability has often been associated with reduced fruit setting and looser bunches [13, 24, 26–28].

Given the strong influence of bunch compactness on fruit quality and susceptibility to disease, identifying and selecting naturally occurring somatic variants with looser bunches has become a promising strategy for improving cultivars with compact bunches, such as ‘Tempranillo Tinto’ [6]. Despite its potential relevance, the developmental and genetic basis of grapevine somatic variants in bunch compactness is known in only a few cases. The RRM somatic variant of the ‘Carignan’ cultivar develops larger and looser bunches due to an increased inflorescence rachis branching and growth, which is associated with a transposable element (TE) insertion that causes overexpression of a homolog of an important plant architecture regulator of the florigen family, the *TFL1A* gene [29]. Similarly, mutations that impair miR396 regulation of *GRF4*, a homolog of important growth regulators in many plants, increase *GRF4* expression, resulting in elongated berry pedicels and ultimately looser bunches in clones of ’Pinot Noir’ [30]. ‘Tempranillo Blanco’, a white-berried somatic variant of the ‘Tempranillo Tinto’ cultivar, carries large somatic deletions and translocations causing sub-haploid gamete lethality, which results in reduced fruit set rate and looser bunches [31].

The VP11 commercial clone of ‘Tempranillo Tinto’ was developed from a somatic variant plant selected for of its loose-bunch phenotype, which is favorable for high-quality wine production [32]. Clonal variation in grapevine bunch compactness has been associated with differences in the bunch rachis or flower architecture [29, 30] or in the number of berries per bunch [33]. In VP11, the development of loose bunches in VP11 is specifically related to a reduction in berry number, which is in turn associated with a reduced fruit and seed set and to much lower pollen viability compared to other clones of ‘Tempranillo Tinto’ (TT) [34, 35]. Gene expression is also clearly affected in VP11 developing flowers compared to other TT clones, which involved the downregulation of gamete formation and pollen-related genes and the upregulation of oxidative stress response genes [35]. However, variant calling from RNA-seq data did not identify any gene affected by high-impact single-nucleotide variants (SNV) or small InDels specific of VP11 compared to other TT clones [35]. The reduced pollen viability and fruit set in VP11 resemble the phenotypes associated with the somatic loss of red fruit color in ’Tempranillo Blanco’ [31], suggesting that as in ‘Tempranillo Blanco’, genome rearrangements might underlie somatic variation in VP11.

To improve our understanding of the developmental and molecular mechanisms leading to variation in pollen viability and bunch compactness in grapevine, we investigated the genome of VP11. A phased genome structural variant (SV) calling using long-read sequencing with Oxford Nanopore Technologies (ONT) detected three inter-chromosomal somatic SV events in VP11. Phenotype and SV segregation studies in a self-cross (S_1_) progeny of VP11 pinpointed one of the somatic SVs as causal for reduced gamete viability. Our findings indicate that heterozygous reciprocal translocations leading to meiosis pairing abnormalities reduce gamete viability, which might decrease the rate of fruit set.

## Methods

### Plant material

*V. vinifera* cv. ‘Tempranillo Tinto’ (TT) clone VP11 was registered in 2006 by the commercial grapevine nursery Viveros Provedo S.A. (VP). The clone was developed by propagation of an old vine (70–80 years old) with loose bunches identified in 2000 in a vineyard located at Elvillar (Álava, Spain). The TT commercial clone RJ51, which was certified in 1990 by the public program of the Regional Government of La Rioja, was used as a control, given that it is one of the most cultivated TT clones in the Rioja DOCa appellation and that it has the type of compact bunches characteristic of TT cultivar. Young leaves of VP11 were collected from a plot of the VP nursery (Varea, La Rioja, Spain, 42.4649007,-2.3940206) as well as from the Instituto de Ciencias de la Vid y del Vino (ICVV) grapevine collection at Finca La Grajera (Logroño, La Rioja, Spain, 42.437448, -2.519582). These VP11 plots correspond, respectively, to two and three cycles of vegetative propagation and grafting from the originally prospected mutant plant. The RJ51 samples were collected from the same ICVV plot. All TT plants had been grafted on Richter 110 rootstocks, were trellised in a double cordon Royat system, and their cultivation was overall similar, following standard practices in the region for TT. Self-cross progenies (S_1_) of VP11 and RJ51 were obtained in 2013 from bagged inflorescences in the VP plot. The 34 individuals of the VP11 S_1_ as well as the 150 individuals of the RJ51 S_1_ were self-rooted, grown in pots and planted in the field in 2017 in the ICVV experimental plot at Finca Valdegón (Agoncillo, La Rioja, Spain, 42.465017, -2.294807). All S_1_ plants were trained in a single cordon system, with a spacing of 2.6 m between rows and 45 cm between consecutive plants in a row.

### Phenotyping

#### Pollen viability

The first inflorescence from randomly selected shoots of three plants of the parental VP11 and RJ51 clones, the 28 VP11 S_1_ individuals producing inflorescences, and ten RJ51 S_1_ individuals were sampled early in the morning at full bloom stage, the time when 50% of flower caps had fallen (E-L 23 stage [36]), . Pollen viability was recorded in years 2021, 2022 and 2023 in all cases except for the RJ51 S_1_ population that was only recorded in 2023. Inflorescences were maintained at room temperature until analyzed within the sampling day in the laboratory. Closed flowers (from inflorescences with recently opened flowers) with erect stamen filaments and yellow anthers from the top, central and bottom sections of the inflorescence were collected and stained following with a modified Alexander’s stain [34], which differentiates between non-viable and viable grains depending on the integrity of the pollen cytoplasm. Five flowers from these three sections were gathered and immersed in 40 µL of staining solution and shaken vigorously for 15 secs to facilitate pollen grains release from the anthers. Afterwards, 20 µL of the solution were transferred onto a pre-heated microscope slide and examined under a Zeiss SteREO Discovery V20 stereo microscope. Photographs of stained pollen grains were obtained with a Zeiss AxioCam camera. Image contrast and saturation were adjusted with AxioVision software (v. 4.8, Zeiss) to facilitate the differentiation between viable (dark blue) and non-viable (light blue) pollen grains. For each sample, three replicates were prepared from independent staining of different flowers, counting an average of about 1,000 pollen grains in each one. Images were processed using the *‘Pollen Counter’* macro [34] in the Fiji (ImageJ) software [37].

#### Seed number per berry

To determine the number of seeds per berry, three bunches per individual were harvested at maturity from twelve VP11 plants and ten RJ51 plants, as well as from the 17 VP11 S_1_ individuals that produced bunches in at least one phenotyping year, and from 27 RJ51 S_1_ individuals in two consecutive years (2022 and 2023). Less than three bunches were used from some S_1_ individuals with lower number of bunches per vine. From each bunch, ten berries were collected from various regions of a bunch (two berries from the top left, and two from the top right sides, two from the middle external and two from the middle internal faces, and two from the bottom position of the bunch). Grapes were opened using a scalpel. The number of seeds was counted and normalized by the sample size (10 berries) to estimate seed number per berry.

#### Bunch compactness

At harvest time in the years 2022 and 2023, bunch compactness was assessed by visual inspection in ten VP11 and RJ51 plants, in the 17 VP11 S_1_ individuals producing bunches, and in 105 RJ51 S_1_ individuals. Bunch compactness was graded by at least two trained inspectors according to the OIV 204 descriptor, which uses an odd-numbered scale with five different phenotype classes from 1 (very loose) to 9 (very compact) [38]. All the bunches present in the plant were considered for compactness grading.

#### Flower sex type

Flower sex was visually determined in 2021, 2022 and 2023 depending on the development of male and female sexual organs according to the OIV 151 descriptor [38] in all inflorescences of ten VP11 and RJ51 plants as well as all VP11 and RJ51 S_1_ progenies producing inflorescences.

### Genomic DNA extraction

Young leaves were stored frozen at -80 °C. Genomic DNA was extracted from frozen leaves using the NZY Plant/Fungi gDNA Isolation kit (NZYTech Genes & Enzymes, Lisbon, Portugal) following the manufacturer’s instructions. DNA was quantified with a Nanodrop 8000 Spectrophotometer (Thermo Fisher Scientific, Wilmington, USA). For genotyping, DNA was diluted to 1 ng/µL and stored at -20 °C. For whole-genome sequencing (WGS), genomic DNA was not diluted.

### SSR analysis

The genetic origin of all VP11 and RJ51 S_1_ individuals was confirmed with seven nuclear short sequence repeat (SSR) loci (*VVS2*,* VVMD5*,* VVMD7*,* VVMD27*,* VVMD32*,* VrZAG62 and VrZAG79)* in a single multiplex polymerase chain reaction (PCR) following an established protocol [39]. Flower type was genotyped with the *VVIB23* marker, which is linked to the sex determination locus [40].

PCR products were mixed with 20 µl of highly deionized (Hi-Di) formamide and 0.2 µl of GeneScan-500 LIZ size standards (Applied Biosystems, Foster City, CA, USA), then denatured at 95 °C for 5 min. PCR fragments were separated on an ABI 3130XL genetic analyzer by capillary electrophoresis at the Centro de Investigación Biomédica de La Rioja (CIBIR). Fragment sizes were determined using GeneMapper v.4.1 (Applied Biosystems, Darmstadt, Germany). Each run included a TT positive control and a non-template sample as negative control. All S_1_ individuals showed profiles compatible with selfing progeny of TT.

### Whole-genome sequencing with ONT long reads

Genomic DNA from the VP11 clone was size-selected to enrich fragments longer than 10 kb using the PacBio Short Read Eliminator (SRE) kit (Pacific Biosciences of California, USA), following the manufacturer’s protocol. As library input, 6 µg of size-selected DNA was used. The VP11 sequencing library was prepared through a ligation-based procedure, employing the Oxford Nanopore Technologies (ONT) SQK-LSK110 kit. The protocol included the NEBNext FFPE Repair Mix (NEB, M6630) and NEBNext Ultra II End Repair/dA-tailing Module (NEB #E7546) from the NEBNext Companion Module for ONT ligation sequencing (New England Biolabs, NEB) for DNA repair and end-preparation. Agencourt AMPure XP beads (Beckman Coulter #A63881) were used for cleanup during library prep. The Long Fragment Buffer from the ONT kit was used for cleanup after ligation. NEB Quick T4 DNA Ligase was used for ligation as described in the ONT protocol.

DNA concentration was measured after every stage with a Qubit 2.0 fluorometer (Thermo Fisher Scientific, Wilmington, USA). For sequencing, 750 ng of library were loaded in one R9.4.1 ONT Minion flow cell. VP11 WGS was carried out at ICVV with a MinION Mk1C sequencer (Oxford Nanopore Technologies, Oxford, UK) following the manufacturer’s protocol. After 45 h, the flow cell was washed using an ONT Flow Cell Wash Kit (EXP-WSH004) and another 320 ng of the same library (that had been stored at 4 °C) were loaded to complete a sequencing run that lasted in total 72 h. For the RJ51 clone, ONT reads were generated in a similar manner at the Max Planck Institute for Biology Tübingen using three SQK-SLK109 and two RAD004 library preps, and five ONT MinION R9.4.1 flow cells (raw reads under ENA accession number PRJEB97948).

### Structural variant (SV) analysis

ONT raw signal was basecalled from VP11 and RJ51 samples using Guppy v5.0.7 in super-accurate mode with the config file “dna_r9.4.1_450bps_sup.cfg” (https://community.nanoporetech.com/). Adaptor trimming was conducted using porechop v0.2.3 (https://github.com/rrwick/Porechop), with default settings. NanoFilt from the NanoPack package [41] was run to discard reads of length < 1 kb and Phred quality < 9 with options: -q 9 -l 1000. Read metrics were estimated using the NanoComp tool from NanoPack and seqkit v0.12.0 [42].

Trimmed ONT reads were mapped to the diploid ‘Tempranillo Tinto’ genome assembly (https://figshare.com/s/c95fa53bb881c55cfefe) [43], with haploid phases inherited from the paternal ‘Albillo Mayor’ and the maternal ‘Benedicto’ parent [44]. This haplotype-resolved reference is therefore suitable for the detection of phased structural variation in a highly heterozygous background. ONT reads were aligned to the TT diploid assembly using *minimap2* [45] with options recommended for ONT reads to run *sniffles*: *-a -x map-ont -Y --MD*. The output of *minimap2* was sorted and saved in bam format using *samtools sort* command [46]. Large SVs of ≥ 1 kb and supported by > 3 clipping reads were called using *sniffles* v1.0.12 [47] from the bam alignment files with options: *-s 3 -l 1000 -d 1000 --max_num_splits 4 --genotype --min_het_af 0.25 --cluster --cluster_support 2 -q 20 -r 1000*. The VP11 and RJ51 corresponding VCF files from *sniffles* were compared using the *merge* tool of *SURVIVOR* v1.0.7 [48] to identify VP11-specific variants as somatic SVs.

### SV validation and segregation analysis

#### Validation and segregation of SV breakpoints

The translocation and duplication SV breakpoints identified with *sniffles* from ONT genome sequencing were corroborated by visual inspection of ONT read alignments to the TT genome assembly in the IGV v2.6.3 viewer [49]. Primer pairs were designed to amplify breakpoint fragments of about 1 kb. Additional primer pairs were designed to amplify similarly sized fragments of the ancestral chromosome sequences at the breakpoint (Tables S1 and S2). We used the Primer Designing Tool in Geneious Prime 2021.2 software (Dotmatics, California, USA) and Primer3 [50, 51], accessed through NCBI. BLASTn (version 2.15.0+) [52] from the BLAST package for UNIX was used to select candidate primer sequences that correctly aligned to the intended haplotypes (‘Albillo Mayor’ and/or ‘Benedicto’) of the TT genome assembly. Fragments were amplified from the VP11 and RJ51 parents, the 34 VP11 S_1_ individuals and 28 RJ51 S_1_ individuals.

PCR amplification was performed with NZYTaq II DNA polymerase Mix (NZYTech Genes & Enzymes, Lisbon, Portugal) following the standard protocol described in the NZYTaq II 2× Green Master Mix manual. The reactions included 10 µM of primer and 1 ng/µL of template DNA sample. The annealing temperatures were between 55 °C and 59 °C and 30 amplification cycles were carried out. PCR products were visualized by gel electrophoresis (1% w/v agarose), run at room temperature for 30–45 min under a constant voltage of 90 V, stained with GreenSafe Premium (MB132, NZYTech Genes & Enzymes, Lisbon, Portugal). Gels were imaged on a ChemiDocXRS + system and QuantityOne software was used to acquire photos (BioRad, Hercules, CA, USA).

PCR products were Sanger sequenced by Eurofins Genomics (Ebersberg, Germany) or by STABVida (Monte da Caparica, Portugal). The sequences were aligned against the TT genome assembly with Geneious Prime and BLASTN.

#### Validation of Tra1-3 phasing

About 1 kb from the translocation breakpoint, a deletion of 5.8 kb in the ‘Albillo Mayor’ allele compared to the ‘Benedicto’ allele on chromosome 3 was identified in the genome assembly of TT RJ51. This deletion was verified by visualization of ONT read mappings from both RJ51 and VP11 in the IGV viewer. The deletion is positioned within a region sufficiently close to the Tra1-3 breakpoint on chromosome 3 to design primers for an amplicon that includes both the Tra1-3 and the InDel breakpoints. In the ‘Benedicto’ haplotype, the 5.8 kb extra sequence prevents effective PCR amplification. Primers were designed and used for PCR genotyping of the InDel in VP11 and RJ51 as described above for SV breakpoints.

#### SNP segregation analysis at Tra1-3 flanks to validate the reciprocal translocation

To validate the exchange of distal portions of chromosomes 1 and 3 in the reciprocal translocation Tra1-3 of VP11, the segregation of heterozygous single nucleotide polymorphisms (SNPs) of TT was assessed in amplicons targeting sequences far away from the translocation junctions. Tra1-3 individuals of the VP11 S_1_ population were compared to RJ51 S_1_ individuals. Primers were designed as described above to obtain amplicons from 1.0 to 1.6 kb that included TT SNPs. The two amplicons designed on chromosome 3 were separated by 6 Mb in the ‘Albillo Mayor’ haplophase assembly of TT, while 7 Mb separated the two amplicons designed on chromosome 1 in the ‘Benedicto’ haplophase (Tables S1 and S2). SNP-rich regions were identified by comparison of alignments of the ‘Albillo Mayor’ and ‘Benedicto’ haplophases of the TT assembly. PCR products were Sanger sequenced by STABVida (Monte da Caparica, Portugal).

The amplified sequences were verified using BLASTN as described above. SNPs in the amplicons were genotyped with Geneious Prime software to ascertain the haplotype/s inherited by each S_1_ individual. Linkage between the pairs of fragments originally located on chromosomes 1 and 3 that are presumably now on the same translocation chromosome at a > 6 Mb distance after the Tra1-3 event was assessed by chi-square two-tailed tests with the PCR results in VP11 S_1_ individuals.

### Statistical analysis of phenotype data according to genotype groups

The mean of each phenotypic trait (pollen viability, seed number and bunch compactness) was calculated from data across all available years for each individual in selfing populations and parental clones. For co-segregation analysis between each phenotypic trait and somatic genome rearrangements, VP11 S_1_ individuals were classified according to the presence or absence of somatic SVs in the parental VP11 clone.

All the genotype classes were pairwise compared using RStudio software version 2023.12.1.402 [53], . The ‘*ggplot2’* package was employed to create graphical representations of the collected data [54]. The ‘*stat_compare_means’* function from the *ggpubr* package [55], which builds upon *ggplot2*, was used for Student’s t-tests.

Chi-squared tests were used to assess the fit of segregation ratios of SVs and other molecular markers in the VP11 and RJ51 S_1_ populations.

## Results

### Loose bunch development associates with reduced gamete viability in VP11 clone

**Fig. 1:**
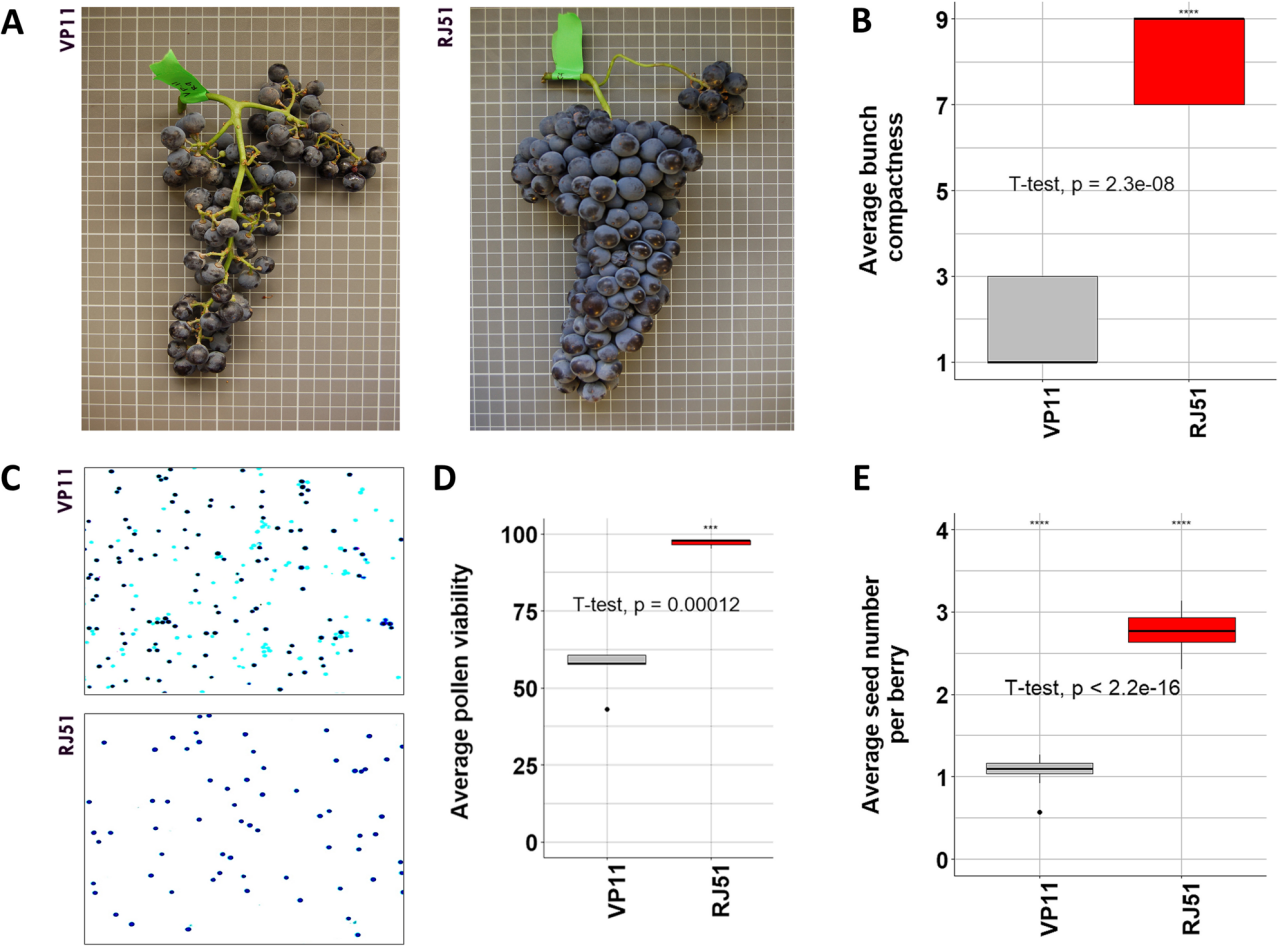
Phenotypic comparison of VP11 (loose bunch) and RJ51 (compact bunch) ‘Tempranillo Tinto’ clones. **A** Images of characteristic VP11 and RJ51 bunches at maturity. **B** Bunch compactness ratings visually estimated according to the OIV descriptor 204. **C** Alexander staining of pollen grains. Viable pollen grains are dark blue-stained and non-viable are cyan-stained. **D** Pollen viability quantification. Viability was analyzed along three years using three replicates of approximately 1000 pollen grains each on each genotype and year. **E** Seed number per berry. Ten berries per bunch were collected to estimate seed number. For bunch compactness and seed number quantification, bunches were collected at maturity, with three bunch replicates per plant in at least 10 plants per genotype for two consecutive years. In box plots, the horizontal black line represents the sample median value, the colored interval indicates the interquartile range showing the middle 50% of scores, and the bars represent lower and upper quartiles. T-tests were used to determine statistical differences between clones

To determine whether the loose-bunch phenotype of the VP11 clone correlates with other reproductive features, we quantified pollen viability, seed number per berry and bunch compactness in VP11, and also in the control RJ51 clone that has compact bunches typical of TT (Fig. 1A).

Over three years, VP11 consistently had low bunch compactness ratings, which ranged from 1 to 3, based on the OIV descriptor 204 [38]. Control RJ51 plants had significantly greater bunch compactness ratings, which ranged from 7 to 9 (Student’s t-test, *p* = 2.3 × 10^− 8^) (Fig. 1B). Estimated average pollen viability was 58% in VP11, compared to 95.0% in the RJ51 control (Fig. 1C and D), confirming the significant reduction in pollen viability reported before for VP11 [34].

Relatively moderate reduction in pollen viability alone, as observed in VP11, would not normally compromise fertilization success in grapevine because several thousand pollen grains are produced per flower, which should be saturating to fertilize the small number of ovules developed per flower (usually four) [56, 57]. As an indirect measure of female gamete viability, we therefore assessed also the number of seeds per berry. The VP11 clone had an average of only 1.1 seeds per berry, significantly fewer than the average of 2.8 seeds per berry in RJ51 (Student’s t-test, *p* < 2.2 × 10^− 16^) (Fig. 1E). The concurrent reduction of pollen viability and seed number in VP11 are symptoms of a global disruption in gamete development or functionality, which could underlie a reduction in fruit set success leading to looser bunches in VP11 [35].

### VP11 has large somatic structural variants including a complex reciprocal translocation

#### Long read genome sequencing and somatic SV detection

While previous variant calling analyses from Illumina short-read whole-genome re-sequencing or RNA-seq data did not detect high-impact SNV or InDel somatic mutations in VP11 compared to other TT clones [35, 58], earlier studies reported that large chromosome rearrangements can reduce gamete viability in grapevine somatic variants [31], as they do in other plants [59, 60]. To evaluate whether large SVs may underlie somatic variant phenotypes in VP11, we performed ONT long-read whole-genome sequencing of the VP11 and RJ51 clones to enable somatic SV calling. About 50× depth read coverage of the haploid grapevine genome was produced from each clone, with ‘clean’ (adapters removed, > 1 kb length and quality > Q9) read length N50 values of 17.7 kb for VP11 and 21.1 kb for RJ51 and average read quality greater than Q14 in both clones (Fig. S1 and Table S3).

**Fig. 2:**
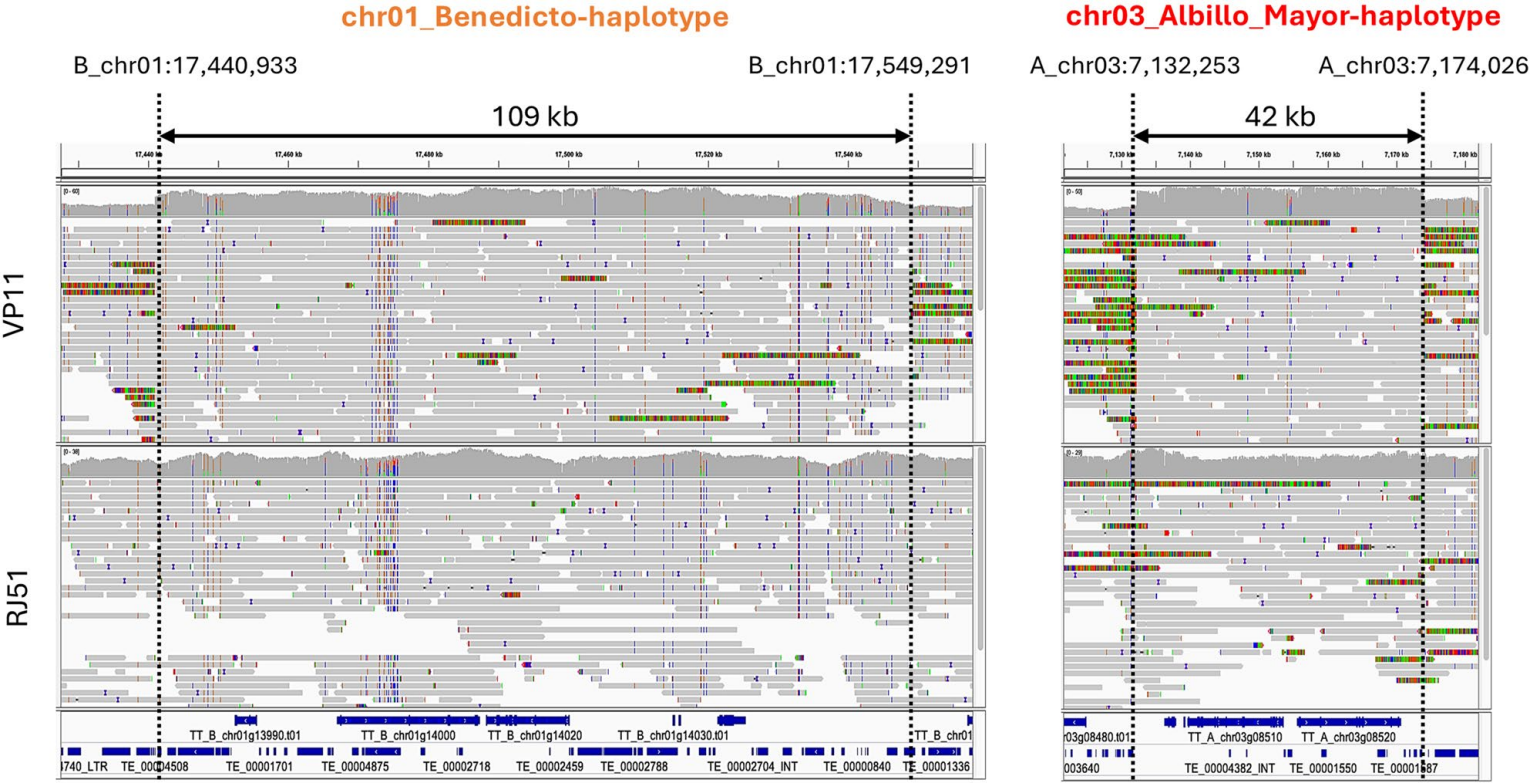
IGV visualization of ONT read alignments supporting Tra1-3 structural variation event specific to VP11 clone. Reads from VP11 and RJ51 clones aligned to the diploid genome assembly of TT cultivar are shown. Soft-clipped ONT reads specific to the VP11 clone support the precise positions of the breakpoints. The structural variation Tra1-3 corresponds to a balanced constitutional reciprocal translocation between chromosomes 1-‘Benedicto’ haplotype and 3-‘Albillo Mayor’ haplotype, with duplications flanking both breakpoint ends. Duplicated fragments are supported by increased mapping depth in VP11, but not in RJ51, as shown in the upper IGV coverage panel. Annotated genes and transposable elements are represented as blue rectangles in the lower section of the image

For phased SV calling, the ONT reads from VP11 and RJ51 were aligned to the diploid RJ51 genome assembly, which includes two haplotypes, one from the ‘Albillo Mayor’ parent and the other from the ‘Benedicto’ parent of TT [43]. Large SV (> 1 kb) calling with *sniffles* and filtering with *SURVIVOR* identified 11 SV calls in VP11 that were missing in RJ51. Because of the length of the ONT reads and the alignment to the diploid assembly, we could readily identify the haplotype phase at each breakpoint. Reads with clipped alignments supporting the breakpoints involved only one haplophase per SV call (Figs. 2 and S2; Table 1), indicating that all VP11-specific SVs were heterozygous events as expected for somatic mutations. Five SVs were insertions or deletions of fragments ranging from 1 to 6 kb size. The other six SVs comprised translocation calls that are paired into three events (Table 1).

**Table 1:** Somatic SV calls detected in the genome of VP11 clone

SV call (Bkpt1-Bkpt2) ^a^	Type ^b^	Length (bp)	Junction	Bkpt1 read support (%) ^c^	Bkpt2 read support (%) ^c^	Bkpt1 TE	Bkpt2 TE	Event ^d^
A_chr03:7132253-B_chr01:17440933	TRA	-	PRECISE	60	48	-	TIR	Tra1-3
A_chr03:7174027-B_chr01:17549291	TRA	-	IMPRECISE	37	45	-	LTR gypsy	Tra1-3
A_chr07:22370961-A_chr11:9266401	TRA	-	PRECISE	70	49	Unknown	LTR	Dup11to7
A_chr07:22370967-A_chr11:9244658	TRA	-	PRECISE	70	24	Unknown	LTR	Dup11to7
A_chr08:20030029-B_chr17:5123987	TRA	-	PRECISE	100	47	-	LTR gypsy	Dup17to8
A_chr08:20030036-B_chr17:5101192	TRA	-	PRECISE	100	34	-	LTR gypsy	Dup17to8
B_chr11:342167-B_chr11:342171	INS	1164	PRECISE	100	100	helitron	helitron	Ins11
A_chr15:4332126-A_chr15:4332133	INS	1842	PRECISE	40	40	Unknown	Unknown	Ins15
B_chr15:10239665-B_chr15:10241077	DEL	-1412	PRECISE	100	100	Unknown	Unknown	Del15
A_chr16:121557-A_chr16:128089	DEL	-6532	PRECISE	49	49	helitron	helitron	Del16
A_chr18:16547881-A_chr18:16547908	INS	1192	PRECISE	35	35	TIR	TIR	Ins18

^a^ Breakpoints specific to VP11 compared to the control RJ51 clone were detected using Sniffles and Survivor tools from ONT reads aligned to the diploid assembly of 'Tempranillo Tinto'. Phased coordinates of joined breakpoints are indicated: A – ‘Albillo Mayor’ haplophase; B – ‘Benedicto’ haplophase. Bkpt – breakpoint

^b^ *TRA* Translocation, *INS* Insertion, *DEL* Deletion

^c^ Percentage of clipping alignments supporting each SV breakpoint

^d^ Grouping of SV calls in genome structural variation events. *Tra* Translocation, *Dup* Duplication, *Ins* Insertion, *Del* Deletion

**Fig. 3:**
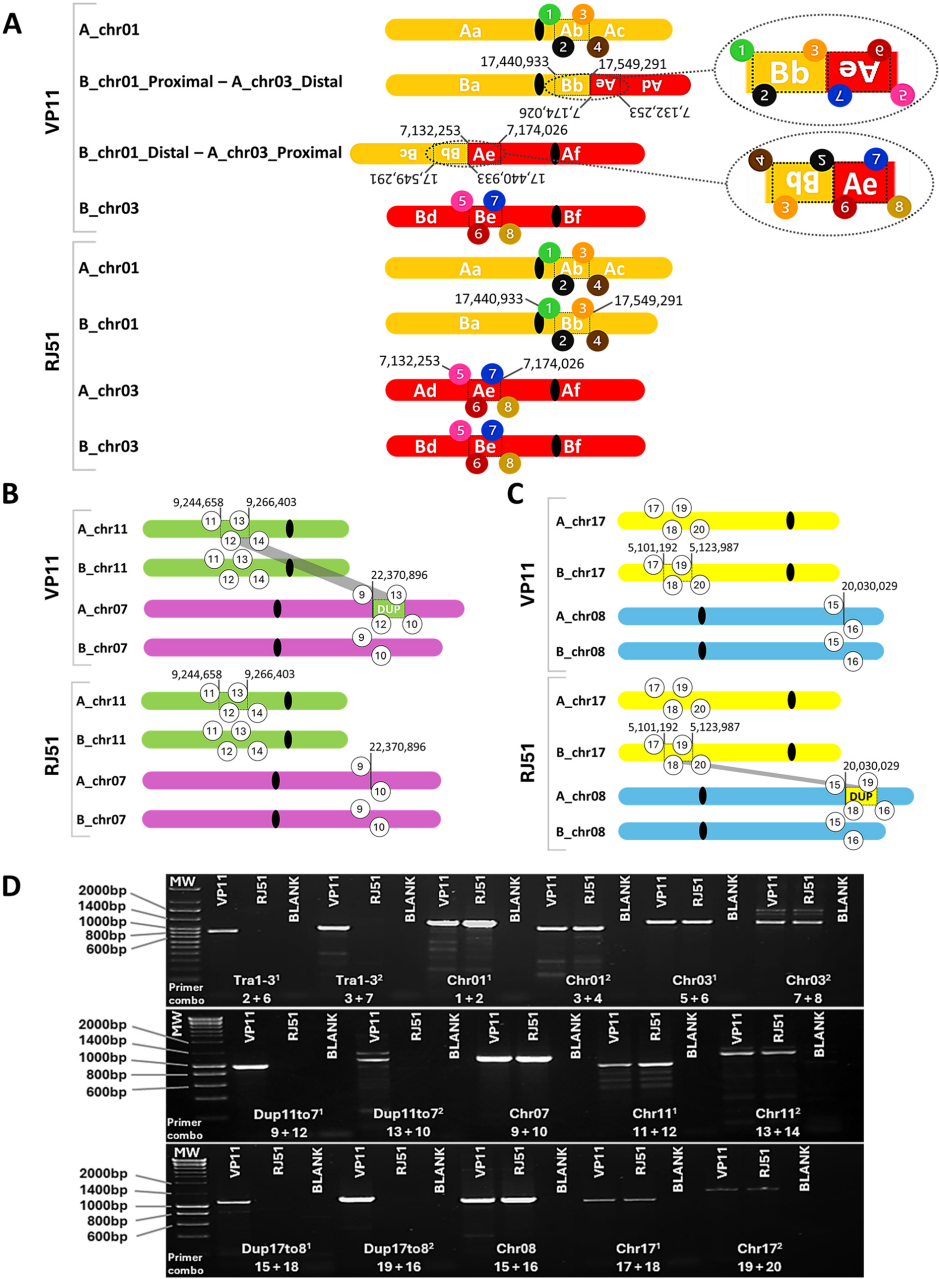
Phased somatic inter-chromosomal structural variation detected in VP11 and PCR validation. Schematic representation of the rearranged chromosomes in VP11 and normal chromosomes in RJ51 for **A** the translocation Tra1-3, **B** the duplication Dup11to7, and **C** the duplication Dup17to8. These structural variation events specific to the VP11 clone were inferred from breakpoint detection using Sniffles tool on ONT genome sequencing reads aligned to the ‘Tempranillo Tinto’ diploid genome assembly. Chromosome numbers and haplotypes (A, ‘Albillo Mayor’; B, ‘Benedicto’; chr, chromosome) are shown on the left for both the wild-type configuration (clone RJ51) and the heterozygous configuration containing the SVs (clone VP11). Lower case letters within the chromosomes indicate different fragments, inversions are denoted by inverted labels. Encircled numbers represent PCR primer positions, which are also inverted if located within inverted fragments. **D** Electrophoresis gel of PCR amplicons generated using primer combinations designed to validate the presence of structural variation breakpoints and the corresponding ancestral chromosomes without breakpoints in VP11 and RJ51 clones. Blank: PCR negative control (no template). The uncropped images of the DNA electrophoresis gels shown in Fig. 3D are presented in Fig. S3A.

One of the three inter-chromosomal events, Tra1-3, is a presumably balanced reciprocal translocation between the ‘Benedicto’-inherited haplotype of chromosome 1 (B_chr01) and the ‘Albillo Mayor’-inherited haplotype of chromosome 3 (A_chr03) (Fig. 3A). Tra1-3 was a complex translocation, since two SV calls with 2-fold increased read alignment depth between them were detected at both chromosome fusion sides (Fig. 2; Table S4). These alignment and breakpoint patterns indicate the presence of large duplications at the translocation breakpoints (Fig. 3A). On chromosome 1, a 109 kb fragment was duplicated: one copy remained in place (chromosome coordinates B_chr01:17,440,933 − 17,549,291) within the 17.5 Mb proximal (centromere-bearing) portion (B_chr01:1–17,549,291), and the other copy was joined to the chromosome 3 breakpoint (A_chr3:7,132,253) as part of the presumably translocated 6.3 Mb distal portion (B_chr01:17,440,933 − 23,766,831) (Fig. 3A). Similarly, on chromosome 3, a 42 kb fragment (A_chr03:7,132,253-7,174,026) was duplicated: one copy remained in the 14.3 Mb proximal portion (A_chr03:7,132,253 − 21,447,870), and the other copy was joined to the chromosome 1 breakpoint (B_chr01:17,549,291) as part of the translocated 7.2 Mb distal portion (A_chr03:1–7,174,026). In the alignments to the diploid assembly, each Tra1-3 breakpoint showed an approximately 1:1 ratio of either soft-clipped reads supporting the breakpoint allele or unclipped reads of the ancestral haplotype supporting the duplication (Fig. 2; Table 1). No ancestral read (same sequence than the haplophase assembly where the read is aligned) or clipped read (read supporting the breakpoint) spanned both breakpoints delimiting duplicated fragments at each end of the Tra1-3 translocation (Fig. 2; Table 1). The lack of ancestral reads spanning the two breakpoints indicates that the SV is fixed in all cells, as no read for the ancestral (non-arranged) allele for the whole region was observed in VP11. The lack of clipped reads spanning the two breakpoints at each side of the translocation supports the duplication of the fragment delimited by the two breakpoints in the other end of the translocation rather than its simple transposition. Together with the twofold increase in read coverage of the duplicated haplotype (Table S4), these results indicate that Tra1-3 is fixed in both meristem cell layers (L1 and L2) of VP11, since the sequenced DNA was obtained from leaf, an organ composed of cells that derive from both meristem layers in grapevine [4].

The breakpoints of the other two VP11-specific inter-chromosomal events, Dup11to7 and Dup17to8, also delimited duplicated fragments showing approximately twofold increased alignment depth (Table 1; Table S4). Soft-clipped alignments at both ends of reads mapping to these duplicated fragments supported the transposition of the duplicated copy to another chromosome (Fig. 3; Fig. S2). In Dup11to7, a 22 kb fragment from chromosome 11 of the ‘Albillo Mayor’ haplophase was duplicated and inserted into chromosome 7, also of the ‘Albillo Mayor’ haplophase (Fig. 3B; Table 1). In Dup17to8, a 23 kb fragment of the ‘Benedicto’ haplotype of chromosome 17 was duplicated and inserted into the ‘Albillo Mayor’ haplotype of chromosome 8 (Fig. 3C; Table 1). Every VP11 read mapping to the duplication-receiver haplophase of Dup17to8 at chromosome 8 had clipped alignments supporting the transposition (Table 1; Fig. S2), indicating that Dup17to8 is fixed in both the L1 and L2 cell layers. By contrast, 30% of the VP11 reads mapping to the chromosome 7 receiver breakpoint of Dup11to7 still supported the ancestral haplotype without the insertion (Table 1; Fig. S2), indicating that VP11 is chimeric for Dup11to7. As expected for somatic SVs, no soft-clipped reads supporting the breakpoints of the three inter-chromosomal events or increased mapping depth in the VP11 duplicated fragments, were detected in RJ51 (Fig. 2; Fig. S2).

Regarding possible functional consequences of the 11 VP11 SVs, only the Dup17to8 breakpoint on ‘Albillo Mayor’ chromosome 8 disrupts an annotated gene, *TT_A_chr08g14410* (Fig. S2B). This gene is the ortholog of the *Vitvi05_01chr08g18520* PN40024 v5.1 grapevine reference gene [61], which is potentially involved in terpene metabolism [62]. The duplicated regions in the three VP11 SVs collectively include 12 annotated genes with increased dosage in VP11 (Fig. 2; Fig. S2; Table S4).

#### Validation and sequence context of SV events in VP11

To confirm the presence of the bioinformatically detected inter-chromosomal SVs, we designed primers to target sequences flanking the Tra1-3, Dup11to7 and Dup17to8 breakpoints. When combining primers against sequences from different chromosomes, they produced PCR amplicons with VP11 genomic DNA, but not with RJ51 genomic DNA (Figs. 3D and S3A). Primers from the same chromosome, as present in the TT ancestor, produced PCR amplicons with genomic DNA from both VP11 and RJ51. The amplification of the ancestral TT allele was expected for the non-variant RJ51 clone, but also for VP11 because it is heterozygous for the SVs and harbors the ancestral and the rearranged sequence at SVs delimiting duplicated fragments (Fig. 2). Sanger sequencing of the amplicons confirmed the presence of recombinant sequences joining fragments that were originally on different chromosomes (Additional Dataset 1).

The analysis of the breakpoint sequence context also identified microhomology of 16 bp and 2 bp at the translocation junctions of Tra1-3, as well as microhomology of the same 7 bp (CCTATGT) at the two transposition junctions of Dup11to7 (Additional Dataset 1). While no transposable element (TE)-related homology was identified between the sequences recombined in the three inter-chromosomal events, the fragments transposed in Dup11to7 and Dup17to9 involved LTR TE sequences, and several of the SVs with length < 7 kb were flanked by helitron or TIR DNA TEs (Fig. 2; Fig. S2; Table 1). Microhomology and TEs may therefore have facilitated some of the somatic SVs of VP11.

#### Validation of Tra1-3 phasing

To validate the haplophases involved in Tra1-3, we exploited a 5.8 kb InDel in chromosome 3 that is absent in the ‘Albillo Mayor’ haplophase but present in the ‘Benedicto’ haplophase (B_chr03:7,224,046 − 7,229,860). This 5.8 kb InDel is located 0.8 kb downstream of the Tra1-3 breakpoint (Fig. S4A-B). A forward primer designed against sequences on chromosome 1 and a reverse primer designed against sequences to the right of the InDel on chromosome 3 were used to amplify a 1.5 kb fragment from VP11 genomic DNA (Figs. S3B and S4C). This result confirmed that ‘Albillo Mayor’ was the haplotype of chromosome 3 involved in the Tra1-3 translocation, since a > 7 kb amplicon would have been expected if the ‘Benedicto’-inherited haplotype of chromosome 3 would have been involved.

#### Inheritance of VP11 inter-chromosomal somatic SVs

The inheritance of the somatic SV events detected in VP11 clone was assessed after germinating selfed seeds of VP11 and growing the plants own-rooted in the field. PCR analysis showed that of 34 VP11 S_1_ individuals, 24 had inherited the Tra1-3 translocation (Table S5). This ratio corresponds to this PCR behaving as a dominant marker for the detection of Tra1-3, with a 3:1 Mendelian segregation pattern for the presence:absence of the translocation (Tra1-3 PCR band) (χ^2^ = 0.55). The inheritance of Tra1-3 breakpoints confirms that the translocation was present in the L2 meristem cell layer of VP11, as gametes in flowering plants are derived from the L2. The inheritance rate also indicates that VP11 is heterozygous for this event. Notably, all VP11 S_1_ individuals carried either both Tra1-3 chromosomes or neither, which indicates that the two translocation chromosomes can only be inherited together (Table S5). These segregation results, along with the decreased gamete viability and number of seed per berry, suggest that in VP11, the only viable gametes are those that inherit a balanced set of chromosomes, i.e., either two normal or two translocation chromosomes, with recombination in the distal parts between normal and translocation chromosomes being possible during meiosis. Nonetheless, this genotyping approach did not enable us to discriminate between heterozygous or homozygous VP11 S_1_ individuals for the inheritance of Tra1-3 due to the duplicated flanks at both translocation breakpoints (primer pairs 1 + 2 and 3 + 4, Fig. 3A).

The somatic duplications Dup11to7 and Dup17to8 of VP11 were also inherited in VP11 S_1_ progeny (Table S5). However, neither Dup11to7 nor Dup17to8 segregated from VP11 clone as expected for the dominant PCR marker in a heterozygote, as these SVs were only inherited by around half of the progeny (χ^2^ Dup11to7 = 0.003; χ^2^ Dup17to8 = 5.2 × 10e^− 06^ for fit to a 3:1 ratio for the presence:absence of the duplication events) (Table S5). All 18 VP11 S_1_ individuals that were PCR positives for the presence of Dup11to7 also carried the ancestral allele without an insertion on chromosome 7 (primer pair 9 + 10, Table S5), suggesting that Dup11to7 was only inherited in a heterozygous state. Similarly, only one out of 14 individuals with Dup17to8 did not carry the ancestral allele without an insertion on chromosome 8 (primer pair 15 + 16, Table S5). The abnormal segregation ratios suggest that Dup11to7 and Dup17to8 are associated with recessive lethal or semi-lethal mutations.

As expected, none of the SVs were detected in S_1_ individuals of the RJ51 self-cross population, and all ancestral TT chromosome sequences were successfully amplified in all of them (Table S6). However, although all the VP11 S_1_ individuals were expected to produce amplification for the amplicons of the ancestral chromosomes 1 and 3 at the Tra1-3 breakpoints (Fig. 3), unexpectedly, six out of ten Tra1-3 negatives and one out of 24 positives for Tra1-3 were also negatives for the amplification of one or more of the amplicons of the ancestral chromosomes without SVs (Table S5). Individuals such as S1-VP11-34, which did not produce amplification using primers for Dup11to7 and Dup17to8 but neither for any of the targeted fragments of the ancestral chromosomes involved (7, 11, 17 and 8) are striking. However, the same DNA sample of S1-VP11-34 amplified both alleles of the SSR markers VVMD7 and ZAG62 (located on chromosome 7) and of VVS2 (located on chromosome 11) (Table S7), indicating that there is no full chromosome aneuploidy in this case, but that the specific chromosome regions affected by somatic duplications in VP11 may be unstable during meiosis.

#### Validation of Tra1-3 as a reciprocal translocation

To validate that Tra1-3 is a reciprocal translocation in which the distal portions of chromosome 1 and 3 have been exchanged, we evaluated the possible co-segregation of SNPs distinguishing the two TT haplotypes in regions that were at hundreds of kb from either side of the translocation breakpoints (Fig. S5). The 1UP and 1DOWN SNP-rich marker amplicons locate 1.2 Mb to the left and 5.2 Mb to the right of the Tra1-3 breakpoint located on chromosome 1 (Fig. S5A). Similarly, the 3UP and 3DOWN regions locate 5.1 Mb to the left and 0.9 Mb to the right of the Tra1-3 breakpoint in chromosome 3. The 1DOWN and 3UP markers thus locate towards the telomeric ends of the distal portions of the translocated chromosomes.

We genotyped by Sanger sequencing of PCR amplicons these four marker regions in the VP11 S_1_ population to test for linked inheritance of regions that were originally on different chromosomes. According to Chi-squared tests of independency, the segregation patterns in the RJ51 S_1_ progeny showed the expected results, with significant linkage between the markers located on the same ancestral chromosomes (1UP-1DOWN χ^2^ = 0.03 and 3UP-3DOWN χ^2^ = 0.004), while the 1UP-3UP (χ^2^ = 0.37) and 1DOWN-3DOWN (χ^2^ = 0.18) combinations were not significant (Table S8). Chi-squared tests returned significant results in the Tra1-3 carrying VP11 S_1_ subpopulation for the following marker pairs: 1UP-1DOWN (χ^2^ = 0.005), 3UP-3DOWN (χ^2^ = 0.007), 1UP-3UP (χ^2^ = 0.001), 1DOWN-3DOWN (χ^2^ = 0.030) (Table S9). These results indicate that the null hypothesis of independent segregation is rejected in VP11 not only for markers originally located on the same chromosome, but also for combinations of markers originally on different chromosomes that become linked due to the reciprocal translocation (Fig. 3A and Fig. S5A). These results support that Tra1-3 is a reciprocal translocation.

### Tra1-3 associates with reduced gamete viability in VP11 progeny

To search for potential causal relationships between somatic SVs and the reduced gamete viability and bunch compactness in VP11, we evaluated the co-segregation of these traits in the VP11 S_1_ population. Because somatic InDels detected in VP11 did not affect genes, we focused on the three inter-chromosomal events.

#### Pollen viability co-segregation analysis

**Fig. 4:**
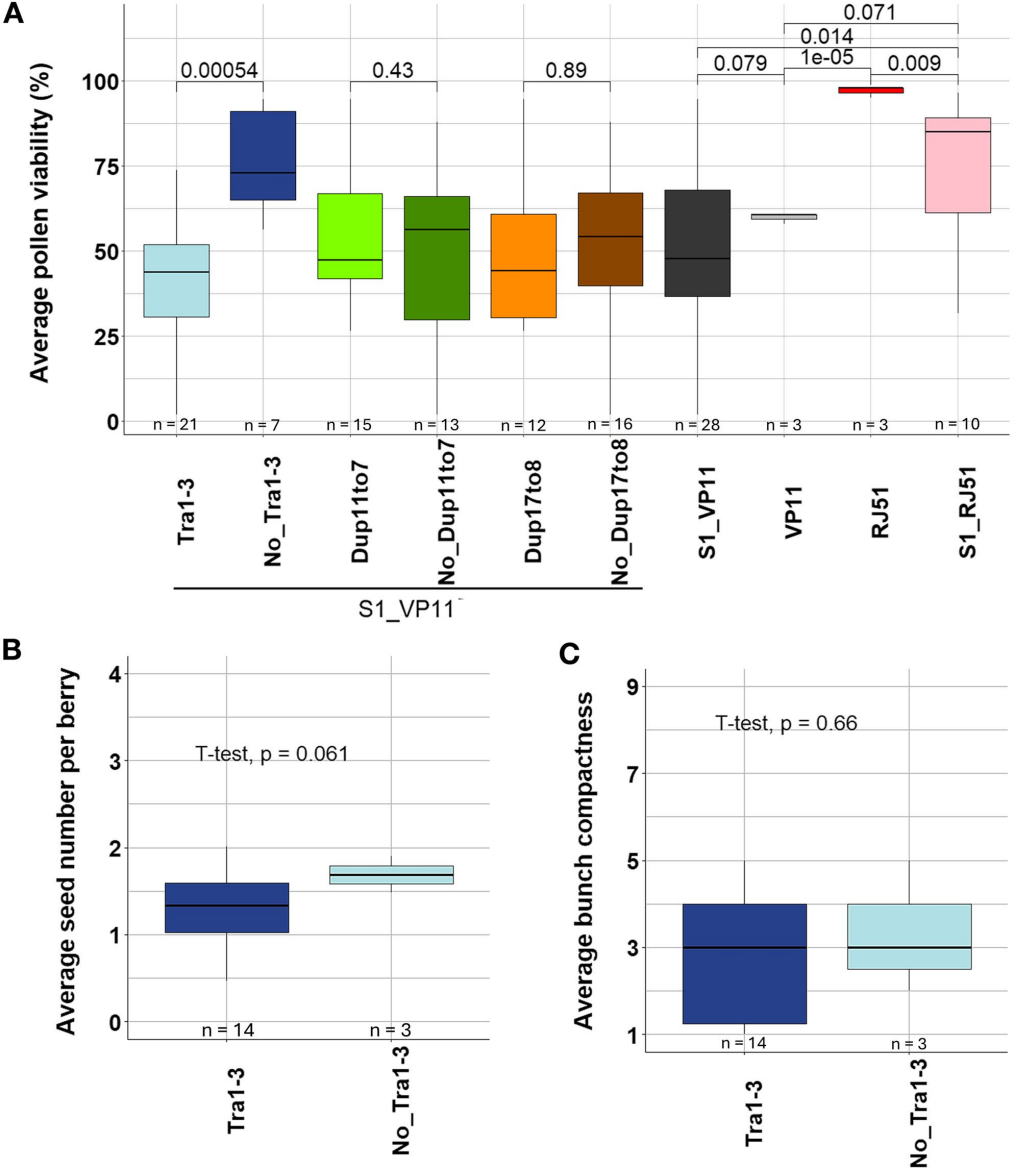
Co-segregation analysis between the presence or absence of Tra1-3 and reproductive phenotypes in VP11 self-cross progeny. **A** Co-segregation analysis of pollen viability with VP11 SV events in VP11 S_1_ progeny and comparison to average values in VP11 and RJ51 clones, as well as to RJ51 S_1_ progeny. **B** Comparison between VP11 S_1_ individuals inheriting and lacking Tra1-3 for the phenotypes of **B**) seed number per berry and (**C**) bunch compactness. For both traits, bunches were collected at maturity, with three bunch replicates per plant. For the S_1_ progenies, data represents single individuals per genotype. In contrast, for the parental clones VP11 and RJ51, at least 10 plants per clone were analyzed, ideally collecting three bunches per plant. In all box plots, the horizontal black line indicates the median value, the colored box indicates the interquartile range (middle 50% of values), and the bars represent lower and upper quartiles. Statistical differences between groups were assessed using t-tests

Over three years, only 28 of the 34 VP11 S_1_ individuals produced flowers in at least one year, with average pollen viability being not different from the VP11 parent (Fig. 4A; Table S5), but significantly lower than in RJ51 and its selfed progeny (Fig. 4A; Table S6). In contrast, pollen viability was significantly higher in RJ51 than the average in RJ51 self-progeny (Fig. 4A; Table S6). In the VP11 S_1_ population, average pollen viability ranged from 1.76% to 94% (Fig. 4A; Table S5), with pollen viability being significantly different between plants with and without Tra1-3 (Student’s t-test, *p* = 0.0005). Pollen viability was on average above 70% in the 7 VP11 S_1_ individuals without the translocation, but on average below 50% among the 21 S_1_ individuals bearing Tra1-3 that produced inflorescences (Fig. 4A). No significant effect on pollen viability was detected for the presence of Dup11to7 or Dup17to8 on VP11 progeny. Together, these results indicate that it is the specific presence of Tra1-3 that is associated with a reduction in pollen viability, despite the possible semi-lethal effects of the two duplications in their homozygous state.

#### Seed number co-segregation analysis

As a proxy of female gamete viability, we assessed the segregation of the number of seeds per berry in mature bunches. Only 17 VP11 S_1_ individuals set fruits and developed mature berries in either of the two phenotyping years, and these were used to study the segregation of seed number and bunch compactness. Unfortunately, the low seed germination and seedling establishment rates of VP11 self-pollinated seeds limited our ability to obtain a larger VP11 S_1_ population for this analysis. Although the statistical power of the comparison was limited because only three of the 17 VP11 S_1_ individuals with available phenotypic data lacked the translocation Tra1-3, there was a trend towards lower seed number in VP11 S_1_ individuals carrying Tra1-3 (mean = 1.30 seeds/berry) compared with those without Tra1-3 (mean = 1.69 seeds/berry). However, this difference was not statistically significant (Student’s t-test, *p* = 0.061) (Fig. 4B; Table S5). The average number of seeds in the VP11 S_1_ population (1.37 seeds/berry) was higher than in the VP11 parent (1.07 seeds/berry) (Student’s t-test, *p* = 0.021 Fig. S6A), which might in part be due to VP11 S_1_ individuals that are homozygous for Tra1-3 and therefore would not suffer from meiosis pairing defects and would not produce unbalanced unviable gametes. This might be the case of the individual S1-VP11-16 that carried Tra1-3 and had high pollen viability and high seed number (Table S5). In contrast, the average number of seeds per berry was lower in the RJ51 S_1_ progeny than the RJ51 parent (1.44 versus 2.76 seeds/berry) (Fig. S6A; Table S6), potentially due to recessive deleterious mutations present in heterozygosity in TT that become homozygous in selfed individuals. No significant effect on seed number was observed for the presence or absence of Dup11to7 or Dup17to8 SVs in the VP11 S_1_ population (Fig. S6A).

#### Bunch compactness co-segregation analysis

Average bunch compactness was significantly lower in VP11 than in the VP11 S_1_ progeny (1.92 vs. 2.94 average compactness score, Student’s t-test *p* = 0.037). There was a tendency towards lower bunch compactness in S_1_ progeny individuals of VP11 with Tra1-3 compared to S_1_ siblings without Tra1-3 (2.86 vs. 3.33 average compactness score), but this difference was not significant (Student’s t-test *p* = 0.66) (Fig. 4C; Table S5). In addition to the mentioned expected absence of Tra1-3 effect in VP11 S_1_ individuals homozygous for the translocation, the statistical power of the comparison is limited because only three VP11 S_1_ individuals lacking Tra1-3 had available bunch compactness phenotype (Fig. 4C, Table S5). Given that bunch compactness is a complex trait, it is also possible that the effect of Tra1-3 or other VP11 somatic mutations was masked by segregating recessive germline alleles at additional loci present in TT that may contribute to this trait. No significant effect on bunch compactness was observed either for the presence or absence of Dup11to7 or Dup17to8 despite the minimum group size went up to 7 individuals for these comparisons (Fig. S6B; Table S5). Bunch compactness was significantly lower in RJ51 S_1_ progeny than in RJ51 parent (5.11 vs. 8.14 average bunch compactness score), while it was significantly higher in RJ51 S_1_ progeny than in VP11 S_1_ progeny (Fig. S6B; Tables S5 and S6). Together, these results suggest that heterozygous recessive alleles reducing bunch compactness segregate in both RJ51 and VP11 selfed progenies, and that additional segregating somatic mutations –Tra1-3 and/or others– further reduce bunch compactness in VP11 progeny.

#### Loss of flower sex type effect on bunch compactness in the VP11 genetic background

As a possibly contributing locus, we analyzed the effect of the flower sex locus on bunch compactness in VP11 and RJ51 progenies. Because TT is heterozygous for the flower sex locus (Tables S7 and S10), selfed progeny will segregate for flower sex type, with female flowers behaving as the recessive phenotype. In both VP11 and RJ51 S_1_ populations, the flower sex marker VVIB23 and flower sex phenotype completely co-segregated, and the flower type showed a Mendelian segregation (3 hermaphrodite : 1 female) (Tables S7 and S10), as expected from the known dominance of the hermaphrodite allele [63]. Among RJ51 S_1_ progeny, female individuals had significantly lower bunch compactness than hermaphrodite individuals (average compactness scores 4.30 versus 5.57; Student’s t-test, *p* = 0.001) (Fig. S7; Table S6). However, no significant differences were found between individuals of the two flower sex types in VP11 S_1_ progeny (average compactness scores 2.33 for females versus 3.27 for hermaphrodites, Student’s t-test, *p* = 0.15) (Fig. S7; Table S5). In addition, bunch compactness was significantly lower in VP11 S_1_ progeny than in RJ51 S_1_ progeny within each sex class, suggesting that segregation of the translocation Tra1-3 or other VP11 somatic mutations may mask the effect of flower sex on bunch compactness, which is typically observed in selfed RJ51 progeny (Fig. S7) and in the progeny of other grapevine cultivars [22, 64].

## Discussion

Somatic variation in grapevine provides a powerful tool for intra-cultivar improvement of critical traits [2, 3, 29]. Among desirable traits, bunch compactness, which is under complex genetic control, is linked to grape production yield, quality, and harvest efficiency [13, 22]. Compared with the large inter-varietal genetic diversity in grapevine, clonal variants carrying only a limited number of somatic mutations can help to identify the mechanisms that modulate this trait [15, 29]. The VP11 clone of the ‘Tempranillo Tinto’ cultivar is a somatic variant with loose bunches associated to a reduction in fruit set [35]. We confirmed that the loose-bunch phenotype of VP11 is stable and associated with reduced gamete viability (Fig. 1), a condition known to decrease fruit set and bunch compactness in grapevine [26, 35]. Through ONT long-read whole-genome sequencing, we identify a reciprocal chromosomal translocation as the likely cause of decreased gamete viability in VP11 (Figs. 2, 3 and 4).

The reduced fruit set and bunch compactness in VP11 is associated with semi-sterility of likely both male and female gametes, as suggested by its lower pollen viability and seeds per berry [35] (Fig. 1), traits that are inherited by selfed progeny (Fig. 4; Fig. S6; Table S5). Reduced gamete viability is known to lower the number of fertilized flowers and thus of setting berries, thereby contributing to a looser bunch structure [16]. This could be the case of VP11 in which only 33% of flowers set berries, which involves a ≥ 1.7-fold lower fruit set rate than in other compared TT clones [35]. Seeded fruit set was only 24% in VP11, representing an even stronger reduction compared with other TT clones [35]. Concurrent reduction in male and female fertility has been reported in other species before, for instance in response to heat stress [65, 66]. Male and female gamete viability are also genetically linked, as shown by the disruption of both ovule and pollen function by gametocidal genes in wheat, as well as by the effect of DEM genes that in *Arabidopsis thaliana* are essential for the viability in both male and female gametophytes [67, 68].

Among less than a dozen somatic SVs detected in VP11 (Table 1), the reciprocal translocation Tra1-3 was a strong predictor of reduced pollen viability in the S_1_ progeny of VP11 (Fig. 4; Table S5). The Tra1-3 breakpoints are inherited and thus not lethal for gametes themselves (Table S5). However, as a heterozygous reciprocal translocation, Tra1-3 should generate about 50% non-viable gametes because of the formation of quadrivalent configurations during meiosis, with adjacent segregation generating unbalanced gametes [69, 70]. These unbalanced “sub-haploid” gametes typically lack essential genomic segments, making them inviable [71]. This mechanism along could explain the approximately 50% reduction in pollen viability and seed number in VP11 (Fig. 1). Somatic reciprocal translocations causing semi-sterility without major effects on vegetative development have been reported in several inter- and intra-specific hybrids. For example, F1 hybrids between watermelon cultivars and disease-resistant wild relatives exhibited markedly reduced pollen viability due to chromosomal divergence, resulting in meiotic pairing defects [72]. Notably, that the two translocated chromosomes were always inherited together in VP11 S_1_ progeny (Table S5) supports the conclusion that unbalanced gametes inheriting only one of the two translocated chromosomes are non-viable.

That there was no statistically significant effect for the presence of the translocation Tra1-3 on seed number or bunch compactness in VP11 S_1_ progeny (Fig. 4) may in part be due to Tra1-3 homozygotes not being expected to suffer from meiosis pairing defects [70, 73], as well as to the influence of recessive alleles segregating in TT cultivar. This is the case of the female allele at the flower sex-determining locus, which was linked to a substantial reduction in bunch compactness in the RJ51 S_1_ population (Fig. S6; Table S6), consistent with findings in other segregating grapevine populations [22, 64]. This effect was weaker in VP11 S_1_ progeny (Fig. S6; Table S5), suggesting that the joint segregation of Tra1-3 or alternative somatic mutations causing the loose-bunch phenotype and sex type may dilute the effect of each individual locus on bunch compactness. The low statistical power resulting from the availability of seed number and bunch compactness phenotypic data in only three VP11 S_1_ progeny individuals lacking Tra1-3 precluded a robust assessment of the effect of the translocation on these traits. Overall, while it is conceivable that gamete semi-sterility caused by the heterozygous reciprocal translocation Tra1-3 could lead to reduced fruit set and bunch compactness [13, 24, 26–28], our results in this small VP11 S_1_ population do not prove this link. Therefore, it is also possible that genetic or epigenetic variation other than Tra1-3 underlies the loose-bunch phenotype characteristic of VP11.

The Tra1-3 SV event linked to reduced gamete viability is a particularly complex translocation as it has large duplicated segments at both translocation breakpoints (Figs. 2 and 3). Replication-based mechanisms have been proposed to generate such configurations, particularly when DNA replication collapses at a replication bubble comprising two replication forks [74]. Similar duplications at translocation breakpoints have been reported in human cancer genomes, such as in multiple myeloma, where they are linked to genomic instability and DNA replication errors [75]. Consistent with the 16-bp microhomology found in one of the Tra1-3 breakpoint junctions (Additional Dataset 1), template switching resulting in duplications and translocations can be facilitated when the replication fork collapses in a DNA break at a sequence with microhomology to another chromosome region [76].

In addition to Tra1-3, ten other large (> 1 kb) somatic SVs were detected in VP11, totaling three inter-chromosomal events and another five InDels (Table 1). The small number of somatic SVs detected in VP11 is in line to what has been reported for two biotypes of the ‘Nebbiolo’ grapevine cultivar and the ‘Tempranillo Blanco’ white grape somatic variant [31, 77]. In contrast, hundreds to thousands of events, often linked to activity of transposable elements (TEs), have been reported for ‘Zinfandel’ and ‘Pinot Noir’ clones, and for bud sports of other grapevine cultivars [78–81]. While technical factors may partly account for differences in these reported levels of somatic SV, the low number detected in VP11 when compared to RJ51 agrees with the recent selection of both TT clones at DOCa Rioja region vineyards. Supporting that both clones belong to a close clonal lineage of the TT cultivar, a clonal phylogeny based on somatic SNVs called from whole-genome re-sequencing data of 50 TT vines identified that both VP11 and RJ51 belong to the ancestral TT clonal genotype group, the group carrying the alleles inherited from the TT parents at the identified somatic SNV positions [58].

Because we had both ONT long reads and access to a phased diploid genome assembly of TT, the varietal genetic background of VP11, we could determine the specific TT haplotypes of the chromosome regions involved in all the called SVs (Figs. 2 and 3; Table 1). This phasing also facilitated the estimation of possible chimerism for the SVs detected in DNA obtained from VP11 leaves, which in grapevine contain epidermal and internal cells that respectively derive from the L1 and L2 meristem cell layers [4]. A 100% frequency of breakpoint reads aligned to the haplophase that is affected by the SV in VP11 at copy-neutral breakpoints, or a ~ 50% frequency at duplication breakpoints like in Tra1-3 (Table 1), indicates that four of the eight detected events were fixed in both the L1 and L2 meristem cell layers. This is consistent with the modest number of L1 versus L2 chimeric mutations detected with HiFi sequencing in ‘Merlot’ grapevine cultivar [82], but contrasts with > 90% layer-specific somatic mutations reported in apricot, where meristems comprise three distinct cell layers [83]. Because Dup11to7 is inherited by VP11 descendants and reads of the ancestral TT haplotype without the transposition insertion were detected in VP11 (Fig. S5; Table 1; Table S5), Dup11to7 should be a chimeric event restricted to the L2, the layer giving rise to gametes in grapevine [4]. The frequency of clipped reads aligned to the transposition-receiver breakpoint of Dup11to7 thus indicates that ~ 70% of cells in grapevine young leaves are L2-derived (Table 1). By contrast, Ins15 and Ins16 may be L1-specific SVs as they were each supported by ≤ 40% of read alignments (Table 1). These values are consistent with the ~ 60% contribution of L2-derived cells to adult oak leaves estimated from somatic mutation analysis [84].

The two duplications detected in VP11, Dup11to7 and Dup17to8, did not individually show significant effects on pollen viability, number of seeds per berry or bunch compactness in the VP11 S_1_ progeny (Fig. 4; Fig. S6 and Table S5). Nevertheless, Dup11to7 and Dup17to8 carrying individuals were underrepresented in the VP11 S_1_ population (Table S5), suggesting a possible recessive deleterious effect on embryo or seedling development. This effect could have contributed to the poor germination and seedling establishment of VP11 S_1_ seeds, which resulted in a small S_1_ population (Table S5). In addition to segregation distortion, the lack of amplification by PCR in some VP11 S_1_ individuals suggests the loss of both ancestral and derived alleles in VP11 progeny at the breakpoints of the two duplications and of Tra1-3 (Table S5). These three events involved duplicated sequences transposed on different chromosomes (Fig. 3; Table S4), a configuration that may facilitate non-allelic homologous recombination (NAHR) and thereby promote deletion of alleles and unequal crossing overs [76]. Other undetected genetic or epigenetic defects in VP11 might contribute to the observed genome instability, giving rise to the observed somatic duplications and translocation, and possibly also to additional genome rearrangements during sexual reproduction. The duplications and microhomology at the breakpoints of different SV events in VP11 (Figs. 3 and Additional Dataset 1) suggest that DNA replication or repair might be defective in this clone. In any case, Dup11to7 and Dup17to8 do not affect the viability of haploid gametes, which can be inferred both from the inheritance of these SVs and from their lack of significant effect on pollen viability in the S_1_ progeny of VP11 (Fig. 4; Table S5). These results are in line with Tra1-3 being the primary cause of the reduced gamete viability in VP11.

## Conclusions

Apart from advancing our understanding of somatic variation for gamete viability and bunch compactness in grapevine, our study demonstrates the power of long-read sequencing combined with diploid genome assemblies to accurately detect SV in highly heterozygous organisms. The loose bunch VP11 clone of ‘Tempranillo Tinto’ cultivar provided an opportunity to investigate the consequences of reciprocal translocations, an event that has not been characterized in earlier grapevine SV studies that mostly focused on inter-varietal and inter-specific diversity [81, 85–87]. Our findings illustrate that complex genome rearrangements that impair meiosis can decrease gamete viability. While long-range genome rearrangements compromising reproductive development would be purged in sexually propagated organisms, they can be selected for intra-varietal improvement and diversification in vegetatively propagated crops like grapevine. The potential utility of heterozygous balanced reciprocal translocations to reduce seed and fruit set without affecting vegetative growth warrants further investigation.

## Supplementary Information

Supplementary Material 1. Additional Dataset 1. Sanger sequences of VP11 breakpoint join amplicons.

Supplementary Material 2. Supplementary Table S1. Primer sequences. Supplementary Table S2. PCR primer combinations and expected amplicon sizes. Supplementary Table S3. Long-read whole-genome sequencing summary. Supplementary Table S4. Read mapping depth supporting somatic duplications in VP11. Supplementary Table S5. Segregation of structural variation breakpoints and reproductive-related phenotypes in VP11 and VP11 S1 progeny. Supplementary Table S6. Lack of segregation of structural variation breakpoints and segregation of reproductive-related phenotypes in RJ51 and RJ51 S1 progeny. Supplementary Table S7. SSR marker segregation in the VP11 S1 population. Supplementary Table S8. Analysis of co-segregation between chromosomes 1 and 3 by PCR genotyping in the RJ51 S1 population. Supplementary Table S9. Analysis of co-segregation between chromosomes 1 and 3 by PCR genotyping in the VP11 S1 individuals carrying the Tra1-3 translocation. Supplementary Table S10. SSR marker segregation in the RJ51 S1 population.

Supplementary Material 3. Supplementary Figure S1. Summary of ONT whole-genome sequencing reads produced for VP11 and RJ51 Tempranillo clones. Supplementary Figure S2. IGV visualization of ONT read alignments supporting Dup11to7 and Dup17to8 interspersed duplications specific to VP11 clone. Supplementary Figure S3. Uncropped images of the DNA electrophoresis gels used for molecular validation of structural variants. Supplementary Figure S4. Validation of Tra1-3 phasing by genotyping a 5.8 kb insertion polymorphism at the Tra1-3 breakpoint region in chromosome 3. Supplementary Figure S5. Design of PCR-based analysis of co-segregation between chromosome 1 and 3 proximal and distal segments resulting from Tra1-3 translocation. Supplementary Figure S6. Co segregation analysis between the presence or absence of VP11 structural variation events and reproductive-related phenotypes in VP11 self-cross progeny. Supplementary Figure S7. Effect of flower sex type on bunch compactness in VP11 and RJ51 self-cross populations.

## Data Availability

ONT raw sequencing data for this study have been deposited in the European Nucleotide Archive (ENA) at EMBL-EBI under accession number PRJEB97948. All other data generated or analyzed during this study are included in this published article and its supplementary information files.

## Abbreviations

Dup11to7: Duplication of a chromosome 11 fragment inserted into chromosome 7
Dup17to8: Duplication of a chromosome 17 fragment inserted into chromosome 8
ONT: Oxford Nanopore Technologies
PCR: Polymerase Chain Reaction
RT: Room Temperature
S1: Self-cross progeny / Selfing progeny
SNP: Single Nucleotide Polymorphism
SRE: Short Read Eliminator
SSR: Short sequence repeat
SV: Structural variant
TE: Transposable element
TT: Tempranillo Tinto
Tra1-3: Reciprocal translocation between chromosomes 1 and 3
VP: Viveros Provedo
WGS: Whole-genome sequencing
