# Supplementary material for: A complex reciprocal translocation is linked to reduced gamete viability in a loose-bunch grapevine somatic variant
Source: BMC Plant Biol. 2026 Jan 27;26:348. doi: 10.1186/s12870-026-08212-7 (PMC12918644; doi:10.1186/s12870-026-08212-7)
Supplement: Supplementary file 1 — Supplementary Material 1. Additional Dataset 1. Sanger sequences of VP11 breakpoint join amplicons. [file 12870_2026_8212_MOESM1_ESM.docx]

**SUPPLEMENTARY DATASET 1**

**A complex reciprocal translocation underlies reduced bunch compactness in a grapevine somatic variant**

Noelia Alañón-Sánchez^1^, Yolanda Ferradás^1,2^, Ilja Bezrukov^3^, Detlef Weigel^3,4^, Pablo Carbonell-Bejerano^1*^, Javier Ibáñez^1*^

^1^ Instituto de Ciencias de la Vid y del Vino (ICVV; CSIC, Gobierno de La Rioja, Universidad de La Rioja), 26007 Logroño, Spain

^2^ Current address: Facultade de Bioloxía, Universidade de Santiago de Compostela, 15872 Santiago de Compostela, Spain

^3^ Max Planck Institute for Biology Tübingen, 72076 Tübingen, Germany

^4^ Institute for Bioinformatics and Medical Informatics, University of Tübingen, 72076 Tübingen, Germany

* Corresponding authors: [pablo.carbonell@icvv.es](mailto:pablo.carbonell@icvv.es), [javier.ibanez@icvv.es](mailto:javier.ibanez@icvv.es)

**Supplementary Dataset 1. Sanger sequencing of VP11 breakpoint join amplicons.** Breakpoints called in VP11 for Tra1-3 translocation and Dup11to7 and Dup17to8 duplications were validated by PCR and Sanger sequencing. The Tra1-3 amplicon included a 5.8 kb deletion confirming the involvement of the ‘Albillo Mayor’ haplotype of chromosome 3. Sequences corresponding to the normal chromosome regions at these loci were verified in both VP11 and RJ51 clones.

**Tra1-3 breakpoint-1:**

***> VP11 gDNA +*** ***B_chr01_1R (2) +*** ***A_chr03_1R (6) primers***

***Sanger sequence color code (according to original positions in the TT genome assembly):***

**B_chr01: 17,441,364 – 17,440,921**

**Common bases between chr01-chr03 breakpoints**

**A_chr03: 7,132,252 – 7,132,688**

GGTAGAATCCTAAATGAAGTAGGCTAATGATGTGACAAGCGAACAAAGGAAGGTTGAGTAAAATTTTGAGGAGGAGAATGACATTAGCCTACCACGATTTAATGGGATAATAGTCTCATTATTTAATGGGGCCATGTCACTCATGGATGCTGTAGGAGGAATAAGAAAATTGAATAGTTGATGATTATGACAATTAATTGATTAAAAACTAGGGTAATGATAAGAAAAGTGGATATCTAGTCTTTGGTGATTTATTTGAAGTGAATCAAAGTTGGTTTAGAGTGCGTTTGGTAGTGGTTTTAGGGAGTGTTTCTAGACTTTCTAACATTTGAAATTTTTTATCTTTCAAGTATTAGAAATGTTAGAAACACTTCCTAAAATCGCTACCAAACACACTCTTATAGTGATTCCAAAAAACGCGTCTAATATTCTTTGATACTCGAAATTCACTTTTAGAAGGATATTTTTTTAAGAGGAGTATGAAGTTTGGAACCGCACACTGATTTAGAGTTGGTGAGGTCAGTTGTAAACCAAAGGTTAACATCAAGATATTATATGTCCCTAGGAGAAACCTCGGTTGAATGGAGAAGTAAGAAGCAAGGGGTCATGGCTAGATCAAGTACAAAGGCAGAGTTCAGGTCTAGGCATATGAGCATCTTTGGTTGCAGATTATTCTTAATGATCTTAAAATTAAACAGGAACAAATAATGCAATTGTATTGTGACAACAAGTCATTGCACAAAATCCTATCCAATATGATCATATAAATCATGTGGATGTTGATAGACATTTCATCAAGCAAAGTCGGATAGTGACTTGATTTGCACTCCCTGTTTTTCAGTGGATAGACAAATTTGGCCAAA

**Tra1-3 breakpoint-2:**

***> VP11 gDNA +*** ***B_chr01_2F (3) + A_ch03_2F (7) primers***

***Sanger sequence color code (according to original positions in the TT genome assembly):***

**B_chr01: 17,548,831 – 17,549,290**

**Common bases between chr01-chr03 breakpoints**

**A_chr03: 7,174,024 – 7,173,648**

TTCATACTACTGAGAAATAAAAGTTTTAGCCAATCATCCTCTGAGGAAAAGCCCTTAGAGGCTGTTTGGCTACTAAGAAAATAGAAACGGGAGAGCTCTGTAAATATTCTAAGTGTAAAACGTAACCTATGTTCTATATCTTTCAAGAGGTGATTTCCACCCCTTATATAGTCTTTACAAAAGCAAAAGCTTGTGATTGGTTAGTTACAAGGATAAGAAAGGAAATTACATCACAAAATACAAAGGAAAATATCTAAAGTTGAGTCGGCAAAAGCAAAGGAAAATTCGCAACACGCATGGGTTATGCGAATTTTGAAGGTGTTATGCGAATTTCGCATAACCTGGAGCAGTTGGCTTCCGAAGGCCATATCTTCCTCATTTCAGCTCCAAATCGTACACGGTTTGAAGCGTTGGATTCTTGACTTCCAGAGCTTTGAAATGGTATATAGTATGTAAAAACTTTTTAAAACCATTAATTTTTTTTTTCATCAATTTCAAACAAAATATGAACTTCCAAAACACCTTCCTTTTTTTTTTTTTCTTTTTTTAACCCGCACCCCTAATTTTACTAACAAAACCCCAAAAAAAAAAAATTAAAATATAAGGTTTTCTTAGAAAAAAAAAACCAAAAACTTAAAAAAAAAACTTTGGGAAAAAAAAAAGGGGGGAGGGAATTTCTTTATTCCCTTAAACAAAGCCTTTTTTTTTTAAATCAGGGGGGGGCCACAACCCAGGGGGTCCATTTTGGATAATGAAACCCAACCCCACGGGCCAAAAAATAATTTTTTNNGAATAAAAAAAGAAACCCTTTCCAATTCNAGTGGCTTNATTTG

**Chr01 ancestral haplotype at breakpoint-1:**

***> VP11 gDNA +*** ***B_chr01_1F (1) + B_ch01_1R (2) primers***

***Sanger sequence color code (according to original positions in the TT genome assembly):***

**B_chr01: 17,440,258 – 17,441,296**

CTACTACATNGGNTGGTAGGCTATCAAAACTAACAAAAAGTATTATTTTGTTGAGTTAAAGTGGGTGAATCCAAGAGGCCATTTACCCTTTTATACTCGTGTAATCATAATCCCTTATTTTCTTCATGTAAACTAAAATGGTATTAATTCTTTTGATAAAATTTAATACTTATTACTTAATAATTTAAGTCGATCTTAAGTTAAATTATATTTAAGTTATCGACTTAAAACTTATTATATAATTTTTACATTAAGTATTAAGGTTGTTTGATAAAATTAACTTAAAATTTATTTTAAATCATCAAATGGACATATTTATTTTCATAAATTATAATTTGGACAAACGAAGTCAAGTAACAATGGAGGTCGTATAGTAATAAAAGAGATGGTGAAAGAAATTAGGACAAATAACGGTAAAAAATAAAATAAAAATATGGATTTAAAAATAAATTAATTATTTTTACTTATTACTTAAAGTTATTTTTAACTTTAAGTCAAGTCATTAAATTATTTTACCAAATATACATAATTTAGTTAATGACTTAAATTAAGTTATTAAGTCACCTTAAGTTATTAAATCATTTTAAGTTATTAAGCTGGTTTAGCAAACACCTATTAACAATAATTTTAGAAAGTGTTTTGAAAGAAAAAAAAATCCAAGNTTTAAAAATAATTAGACCGCCGTTTTTTGGAATCACTAATAAGAAGGGGGGTTTGGTTAGCGAATTTAAGAAATGGNTTCTTAACATTTTCTAATACTTTGAAAGAAAAAAAATTTTCAAATGTTAGAAAGGTTAGAAACACTTCCCTAAAAACCACTACCAAACGGCACTCCTAAACCAACTTTGATTCANTTTCAAATAAATCACCAAAGACTAGATATCACTTTTCTTNTCATTTACCTTAGGTTTTAATCAATTANNGCCNNAATCATCAACTATTCCATTTTNTTATTCCTCTACAGCATCATGAGTGACATGGCCCATTAANTNATGGAGACTTANTANNCNTTAATTCGGNGGGTAGGCTANGTCATTC

**Chr01 ancestral haplotype at breakpoint-2:**

***> VP11 gDNA +*** ***B_chr01_2F (3) + B_chr01_2R (4) primers***

***Sanger sequence color code (according to original positions in the TT genome assembly):***

**B_chr01: 17,548,841 – 17,549,790**

CATTCCATACTCTGAGAAACTAAAAGTTTTAGCCAATCATCCTCTGAGGAAAAGCCCTTAGAGGCTGTTTGGCTACTAAGAAAATAGAAACGGGAGAGCTCTGTAAATATTCTAAATGTAAAACGTAACCTATGTTCTATATCTTTCAAGAGGTGATTTCCACCCCTTATATAGTCTTTACAAAAGCAAAAGCTTGTGATTGGTTAGTTACAAGGATAAGAAAGGAAATTACATCACAAAATACAAAGGAAAATATCTAAAGTTGAGTCGGCAAAAGCAAAGGAAAATTCGCAACACGCATGGGTTATGCGAATTTTGAAGGTGTTATGCGAATTTCGCATAACCTGGAGCAGTTGGCTTCCGAAGGCCATATCTTCCTCATTTCAGCTCCAAATCGTACACGGTTTGAAGCGTTGGATTCTTGACTTCCAGAGCTTTGAAATGGTATATAGTATGTAAAAAATGGACTTCGTTAAGTGCTCCAAAAGTGAAAAAGAAGACTGCAGCTGCTGTCCTCTGTTTTCCTCTTCTCCATTGTTCTTTCCTTGCATACTTTGAACGACTTTGGCAAAGGGCTATGGAGCTCCAAAGCTTGGTTCTTCATGAATTTGAGCTTCCAAAAGCTTTGCCATAACTTGTCCAAGTAGCTCCTCCATCATTTGGCATGCTTGAATTGATTCATAAGCTGATAAAAACATGTAAACTTGCCACAAAATGGTTAAAACCAATTACTAAGACCTTAATGAATTAATTGGGTTAAATGAATATGATTACTACTCAAGGTGCTTAAACCATTATAATTAGNCTACAAATACACTTTTTGGTAGAATCATGAGCCTCCAATTTACTTGTACTTATACTTCACTCTATAGTATCCAGCATTANAAATGCATATTTCAGTATGTTCTGTTTTTTNTGCANATGNNACATTATGATTTTGTCATTTCAG

**Chr03 ancestral haplotype at breakpoint-1:**

***> VP11 gDNA +*** ***A_ch03_1F (5) + A_ch03_1R (6) primers***

***Sanger sequence color code (according to original positions in the TT genome assembly):***

**A_chr03: 7,131,521– 7,132,503**

ATCCCAGTAAAAGCAGGTTTATAAAGGGTTTATATAGATCCTAAGACCTAGAGGATCAATACCCAAACTTTCCAAAAGAATAAATATTTAATCTCTAAAAATGTGATCTACCATATTTTACAAAAAATAATTTAAAAACTTAAGAATTTAAAAAAAAATTGAAAAGATTTCATATTTTAAATTAAAAAAGATTTAGAAAAACTTTCTTTTAAAATTTTTTTAATTTAAATTCTTTTTCAAATCTCTTTTCCAAATTCCTTAAAAGATTTGGAAAGATTCTCTTTTTTAAATTCTCAATTTAAAATCGTTTTCCAAATTATTTTTATTATTTGATATATTTTTACTTAATCATTACTGTAGATGATTTGAAGGAAATAGAATGGCTTAGACAACACCTTGAAGTAGAGTTTAAAATAAGAGATCTTACAAGACTAAAGTGTTTCTTGCATTGAAGTGGCCCATTCAAGGAGTGGTATTTTCATATGACAACAAAATATGTTCTTGATTTGCTAAAGGAAACTAGGATGTTAGGATGTTAGCTAATTGACATTGCCATTGAACAAAATCACAAATTTGGTGATGTTAAGGAGGTAGTTGTAGACAGAGGTATATATCAAATTTTAGTTGAAAAGCTAATTTATTTATTCCATACCAGACTAGATATAGCCTATGTCATGAGTAGTGTAAGTCAATTTATGCATTCACTTAGAGAAAGTCATTTTGAAGCAATCTATAGAATTCTTTGATACTCGAAATTCACTTTTAGAAGGATATTTTTTTAAGAGGAGTATGAAGTTTGGAACCGCACACTGATTTAGAGTTGGTGAGGTCAGTTGTAAACCAAAGGTTAACATCAAGATATTATATGTCCCTAGAGAACCTCGGTTGAATGAAGAGTAGAGCANGGTCATGCTAGATCAGTACNAGCAGAGTCAGTCTAGCATTATGAGCATCTTTGTGCAGATTATTCNATGATCTTAAA

**Chr03 ancestral haplotype at breakpoint-2:**

***> VP11 gDNA +*** ***A_ch03_2F (7) + A_ch03_2R (8) primers***

***Sanger sequence color code (according to original positions in the TT genome assembly):***

**A_chr03: 7,173,554 – 7,174,715**

TAATTGTAAGCTTTTAATTAGTTTTGACCAAAATTAACAAAAGAATTGGTTTTAGTTGTTAAATAAAAAAAAATTGATTATAGTTATTAAATCAAATTAAGCCAATCGAAATTGAAAGGGTTTCATTATTAATACATAAAAATTAATTAATTAGACAGTTGAGTTTGATTTCAATACTCAAAATTGAACCCATTGGATCGTGCACACTCGTGATATAATATAAATAAGACTATGATTAATGGAATAAGATAATTTCATACCTCCTTTTTTTATTCACAATGTTTTTTTTTTTAGTTTTTTGTTTTTTTTTTCTTAAGAAAACTTTATATTTAATTAATTATCTATTGAGATTTTGTAGTAAAAGTAAGGGCTGCGGTTAAGAAAAGGAAAAAAAAAAAAAGGAAGGTGTTTTGGAAGTTCATATTTTGTTTGAAATTGATGAAAAAAAAAATTAATGGTTTTAAAAAGTTGAGTTTTTTTTTTTTTAAATAATTGGGAAAATATAATATAATATAAATAGGGTTCTTCTTCCAAAAAAAAAAAGGGGGGTTTTTTTTTATAAGGGGGGATTGAAAAAAAAAGTGGGGGTTTTTTTAAAAAATTTGAAAAAAAAACCGGAAATTTTTTTTAAAAAAAGGTTTTTTTTTAAAAAAAAAAATTTGGGGGTTTTTTTAAAAAAACAAAAGTGGAATAAAAAAAATTCTTGGGGGTTTTTTTTGTTAAAATGGGGAAAAAAAATGAAAAAAAAAAAAAGTGGGGTTTTTTTTTAAAAAGGTTTTTTTTTAAAAAAAAAAAGGGGTTTAATTGTATATAAAAAAAGGGTTAAAATTTGGGGGTTCCATAATTCCGGGGCCTTTTTGGCCACCTGATGGGCCAAAATAGTAATTTTGGCCTTTTCCCCCCTTGGGAGCCTTTGAAGGGTAAAAATTTTCCCCTTTTTGGATAAAGGAAAATTATACCCCTAAAAACTCCAAATATAAGGTTGGGAAAGGGGTTTTTTTAGAAGGGTTTTTAAAGAAAAAGATTTTTTCAAAAAAAATTTTTTTTTTTTTTTTTTTTGTTGGCGGTCCTTTTAGCTTAAGTTTTTTTTTTTTTTTTTAATCTTATTTCTGGAGCACTTTGCTGCAGCATGTTTTAGAGTGGGGACCCGGTTTCCTTCGA

**Dup11to7 breakpoint-1:**

***> VP11 gDNA + A_chr07_1F (9) + A_chr11_1R (12) primers***

***Sanger sequence color code (according to original positions in the TT genome assembly):***

**A_chr07: 22,370,432 – 22,370,969**

**Common bases between chr07-chr11 breakpoints**

**A_chr11: 9,244,650 – 9,245,031**

ATTATAACACTTATCTTAGCACTTAGAGTGATCGACTTCTCCATTTGGCTTATACCATTTGCCACTAATCTTGGCTTAAAGTGATTAACTTCTCCACTTGACTTATACTTGATTTTGTACACTTATTTGATATCAATTAGTTTCTTAAAATTAAGGGGATTGCTTAACTCACATGTACTATTTTAGTCAAATGCATGAATTTTTCATCCATAACTTTAAACCAACAATCATCTTAAGCAACTTCTTCATCACTCAATAGATCATTGTTTACAAATAAAGCAAATTTGAAATTCTTTACATATTGGGTCATTGTCATCACCAACCACATAATCTTGCAAACATGTAGGTAATGCACGTTAATACTTTGGGTGCCTTGATGACCTATAAGACATGAAATCACAATTGGTGGATATTAAGTTGCATATTTTTTTCCTTCACGTTACTCTTTAAGATCGATGCTAGTATTTGATGGTCATTTTTTATTATAAGCTTTTATCACTTGATTATAACCAATAAAAATTCATTTTTCACCTATGTGTGGACCCCGCATTTTCGATGGCTCGAATGCGTTTCCCACTCGAATGGCGAGCTCGATTTTTATTTGAAAAAAATATTTTTATTGATTATTTGAAAATGACTTGGAGTCGCCACTTATTTTTGTTTTATTTTTAAAGGGTAAACAAAATAAGAAAGAAAAACCCTAAGTGTGACTCCTTATTTTAGAAAAGGTGATCTACGAAAACCGGATCGAGTTCGGGGGTCAGGTTACTTATCGGGAAGGTACGGCAAAAACCGTAGCACCCCTCTAAGTCCCTAAAGTCAGGTCTCTACTACTAAAATGAAGCTGACATGGCAATCAATGAGAAAATCAGTAAATACTCAAATCAATCATGCACATATGAAGGGGCAAA

**Dup11to7 breakpoint-2:**

***> VP11 gDNA + A_chr11_2F (13) + A_chr07_2R (10) primers***

***Sanger sequence color code (according to original positions in the TT genome assembly):***

**A_chr11: 9,265,851 – 9,266,400**

**Common bases between chr07-chr11 breakpoints**

**A_chr07: 22,370,960 – 22,371,506**

AGTCTCCTTCTCTCTCAAGCAGCTGGCAGCTCCAGAATCTTTCACAGTCGTCAATGGAATCCCCCCCTCCTTTCTCTCTAATGGAAACTACCTCTAGACTCCCCGGAAAGTAACACCGTCAAACTTCTCTGTTCTCCCTTTCCAGAAAATGCTCTGTTCCCTTCAGCTTGCTTCCACCCTCCTCTCAAAAGCACCACACCCTCTCTCAAGAAAACTCCCTGAAGCCCCTTCCAAAACTCAGTCTCCGACCAGCATCAAAAAGCTCTCCCCCTCTCTCATACGCTCCTCCTGCTGAAAAGGCACCCCTCACTGTTCCTGCAGCTGGCCTGCCACCACCGCAGCTGCTCTGCTATCCAAATAACTGCCTGCAAAATCCCCCCAGCATCCTCCTACAGGTGTCGCCTCTTGATAGCTCCTCAAAAAGCAATAACTGATTCTGTAGCTGCCAATTAAAAGATGCCATGTGGCTCTCTGGGGTTGCGACACTTGGCAAAACAAGCTGCATGAAAATGCATGAGTCCGAGCCCCAAATGGGGTCTACACCTATGTCATCAAGTTTTTGTCGTAATGCATCGTCCCATGCGCCGCATTCCATGATCGAGGCGATGATGTCTCCGAGGGCATAACGACAACCCGAGCTTAGGCTACCACTGTATGAGCATATATTCTGCAATCCAACACCCTTAGATGACTAATGTTCCCATGACTCCAAGCTTCTTATGGGGTATGTAACTTGCTCAAGAAATTGCTTCGATAGAAAGGAATTTAGGCAATTGTTTTCAATGCAACATACACCTCATTGTGTCCATGATTTTTTTTTTCTCTCTACAATTTTATTTTGTTGGCAGATTGTACCTTATAGTCATTTGATGCTCAATATCATATTCTTTTTTTTCCAAAAAAATTTCACAAATGATACATGTAGACCATTATCTATTCTCAAAATTTTAATGGTATGCCCACTTTGTGTTTTCCATATAAGCTTAAAAAAAGATGTCATGTGTATCTTCTTTTTTATTTTTAAAAAATAAACCTAAATCTCCAAACTAAAATCATCATTAGGATGTAATAAAATACATAGATCTCTA

**Chr11 ancestral haplotype at breakpoint-1:**

***> VP11 gDNA + A_chr11_1F (11) + A_chr11_1R (12) primers***

***Sanger sequence color code (according to original positions in the TT genome assembly):***

**A_chr11: 9,244,141 – 9,245,039**

AATGATTGGGAGGTCATTTCCTACTCTGAAACAAACTTTGGAACCCTAGGTTTTGATAGCTCAAGGATTGGATGTACCAAATTTTGTGAAGGATGTAACGAATTTTATAAATGATGTACCAACTTTTGTAAATGATGTACCAAGTGGTCCAAAGTTAGTTTCAAAGTGGTGAACGACCTCTAATCACTTCTTAAGGCTGTCTAAATGAATAAATGGACCATGGAAAGGAAGATAGGTGTTCTGTTAGCTCAAGGATTGGAGGTATCAAATTTTCTTAAGGATGTACCTAATGTTCCAAATTCTGTTTCAAAGTAGGGAACAACCTCCAATCACTTTTCAAAGATCCTTAAAAGAATGAATGGACCATGGTTAATAAGATAGGGGTCCCGGTAGCTCAAGGATTGGATGTACCAATTTTTTTTAAGGATGTACCAAATTTTGTAAAAGATATACCAAAATTTGTAAATGACATACCAAGGTTGCCAAAGTCCATTTCGAGGGGGGAAACAACATCCAATCTGTGGACCCCGCATTTTCGATGGCTCGAATGTGTTTCCCACTCAAATGGCGAGCTCGATTTTTATTTGAAAAAAATATTTTTATTGATTATTTGAAAATGACTTAGAGTCGCCACTTATTTTTGTTTTATTTTTAAAGGGTAAACAAAATAAGAAAGAAAAACCCTAAGTGTGACTCCTTATTTTAGAAAGGTGGATCTACGAAAACCGGATCGAGTTCGGGGGTCAGGTTACTTATCGGGAAGGTACGGCAAAAACCGTAGCACCCCTCTAAGTCCCTAAAGTCGGGTCTCTACTACTAAAATGAAGCTGACATGGCAATCAATGAGAAAATCAGTAAATACTCAAATCAATCATGCACATATGAGAGGGCAAAGATATA

**Chr11 ancestral haplotype at breakpoint-2:**

***> VP11 gDNA + A_chr11_2F (13) + A_chr11_2R (14) primers***

***Sanger sequence color code (according to original positions in the TT genome assembly):***

**A_chr11: 9,265,904 – 9,266,994**

AATGGGAATCCCCCCTCCTTTTCTCTCTAATGGAAACTACCTCTAGACTCCCCGGAAAGTAACACCGTCAAACTTCTCTGTTCTCCCTTTCCAGAAAATGCTCTGTTCCCTTCAGCTTGCTTCCACCCTCCTCTCAAAAGCACCACACCCTCTCTCAAGAAAACTCCCTGAAGCCCCCTTCCAAAACTCAGTCTCCGACCAGCATCAAAAAGCTCTCCCCCTCTCTCATACGCTCCTCCTGCTGAAAAGGCACCCCTCACTGTTCCTGCAGCTGGCCTGCCACCTCACTGCAGCTGCTCTGCTATCCAAATAACGGCCTGCAAAATCCTCTCCAGCATCCTCCTACAGGTGTCGCTTCTTGATAGCTCCTCAAAAAGCAATAACTGATTCTGTAGCTGCCAATTAAAAGATGCCATGTGGTCTCCTGGGGTTGCGACACTTGGCAAAACAAGCTGCATGAAAATGCATGAGTCCGAGCCCCAAAATGGGGGTCTACACAATCACTTCCCAAGGGTCTCTAAAAAAAGAAATGGACCATGGGAAGAGAGATAGGTGTCCCGTTAGCTCAATGATTGGATATATCAAATTTTATCAAAGATGTACCTAGGGTTCCAAAGTCCGTTTCAGAGTAGGGAACAACCTCCAATCACTTCCCAAAGATCCCTAAAGGAATAAATGGATCATGGGTAATAAGATAGAGGTTTTGATAGTTCAAGGATTGTATATACCAAATATTTTTAAGGATGTACCAAATTTGTTGAATGATGTACCAAATTTTGTGAAGGATGTACTAGATTTTGTAAATGACGTATCAAATTTTGTAAATGATGTGCCAAGAGTGCCAAAGTTCGTTTCGAGGTGGAGAATGACCTTTAATCACTACTTGAGGGTCCTCAAAGGATAATATGAACCATGAGAAGATAGATAGGGGTCTCAATAGCCCGAGGATTGGATATACCAAAATTTCTTAACGATGTACCAAATTTCCTTAAGGATGTACCTATGATTCCAAAGTTCATTTCAGAGTAGGGAATGACCTCTAATTACTTCCTAAAGATCCCTAAAGAATAAACAAACCATGAGTATAATA

**Chr07 ancestral haplotype:**

***> VP11 gDNA +*** ***A_chr07_1F (9) + A_chr07_2R (10) primers***

***Sanger sequence color code (according to original positions in the TT genome assembly):***

**A_chr07: 22,370,421 – 22,371,503**

ACTTATGACCATTTAACACTTATCTTGCCTTAAAGTGATCGACTTCTCCATTTGGCTTATACCATTTGCCACTAATCTTGGCTTAAAGTGATTAACTTCTCCACTTGACTTATACTTGATTTTGTACACTTATTTGATATCAATTAGTTTCTTAAAATTAAGGGGATTGCTTAACTCACATGTACTATTTTAGTCAAATGCATGAATTTTTCATCCATAACTTTAAACCAACAATCATCTTAAGCAACTTCTTCATCACTCAATAGATCATTGTTTACAAATAAAGCAAATTTGAAATTCTTTACATATTGGGTCATTGTCATCACCAACCACATAATCTTGCAAACATGTAGGTAATGCACGTTAATACTTTGGGTGCCTTGATGACCTATAAGACATGAAATCACAATTGGTGGATATTAAGTTGCATATTTTTTTCCTTCACGTTACTCTTTAAGATCGATGCTAGTATTTGATGGTCATTTTTTATTATAAGCTTTTATCACTTGATTATAACCAATAAAAATTCATTTTTCACCTATGTCATCAAGTTTTTGTCGTAATGCATCGTCCCATGCGCTGCATTCCATGATCGAGGCGATGATGTCTCTGAGGGCATAACGACAACCTGAGCTTAGGCTACCAGTCATATGAGCATATATTCTGCAATCCAACACCCTTAGATGACTAATGTTCCCATGACTCCAAGCTTCTTATGGGGTATGTAACTTGCTCAAGAAATTGCTTCGATAGAAAGGAATTTAGGCAATTGTTTTCAATGCAACATACACCTCATTGTGTCCATGATTTTTTTTTTTCTCTCTACAATTTTATTTTGTTGCAGATTGTACCTTATAGTCATTTGATGCTCAATATCATATTCTTTTTTTTTTTTCCAAAAAATTTTCCAAAAGGAAACTTGTAAACCTTTCTCTTTTCCCAAAATTTTAAGGGGAAGCCCCCTTTTGGTGTTTCCATAACCTTAAAAAGAGAGGCCATGGGAACCTTCTTTTATTTTTAAAAAAAAAACCAAATCCCCAACTAAAATCCCCTTTAAGGGAAAAAAATAATAAATCCTTTTA

**Dup17to8 breakpoint-1:**

***> VP11 gDNA +*** ***A_chr08_1F (15) + B_chr17_1R (18) primers***

***Sanger sequence color code (according to original positions in the TT genome assembly):***

**A_chr08: 20,029,535 – 20,030,034**

**B_chr17: 5,101,188 – 5,101,777**

TTTGGGGGGGGGGGGGAGGGAAGAGGCAATGGTGAATTGCAAGAGGATGACCAAGAAGAAGCCTGGATATGAGGAGGCCTGGGTGTGAGGAACGGAGAGGACGACGAACATTTATTCCCTAGGTGGCCATTGAGAATTCTCTCTCTCTCTCTCTCTCTTTCTCTATATATTTATCTATCTCTACCAACCCGTCTTAAAAAGGAGAATCCCATCGGTGGCCCCCCTCCAGCCCGATTTTCTCTGGCTCTTTGAAAAACACAACCAGCTCCTTCCCCATGTAGTTTATTTTCCACCAAAACTTTTTTAATCTCCCCTAGAATTTTCATATCTTCTTCCCGACATGTACTACAAAAAAAAAAAAATCTTTAGCATTCCGTATAATATATCTATTTTTGTGATTATTGCAACAAATTAAAATAAACTCAAATTATTCCATAAATATATTAATTCCTCAAATAAACATACAGTAGTTTATTTCTACGTACTTTTGAGTGACGAGGTGTGGACCCCGCATTTTCGATCGCTCGAATGCGTTTCCCACTCGAATGGCGAAACTCGATTTTTATTTAAAAAAAATTGATTTATATTGATTATGAAAATGACTTGGAGTCGCCACTTATTTTTGTTTTATTTTTAAAAGGGTAAACAAAATAAGAAAGAAAAACCCTAAGTGTGACTCCTTGTTTTGGAAAAGGTGGTCTGTGAAAAAACCGGATTGGGTTCGGGGGTCAGGTTACTTATCGGGAAGGTACGGTAGAAGACCGTAGCACCCCTCTAAGTCCCTAAAGACAGGTCTCTACTAATAAAAATGAAGCAATCATGGCAATTGATGAGGAAAAACAATGAATACCTGGAACTATCATGCACATAGGAAGAATCAAAACATGTATATGGAAATACCGGAATGGGAATAAAATACGTACCTGGGTGACGAGCCACAATGCGCTTATCAAAAAGTGAGGTTAGGGCACAATTAAAGAATGATTTCAAGCATGTCGTAGGGCAAAAAAAAAAACAATCATGCATGACAATTAAATCAAACAAACAATCAATCAATCATAAGGAAAATCACATATGTGGGCCCCCCCA

**Dup17to8 breakpoint-2:**

***> VP11 gDNA + B_chr17_2F (19) + A_chr08_2R (16) primers***

***Sanger sequence color code (according to original positions in the TT genome assembly):***

**B_chr17: 5,123,445 – 5,123,980**

**A_chr08: 20,030,034 – 20,030,594**

AAAACCCATACCTGTGGCCCTTAACGTCCACAGCAAGCAATAACCCATATCCATGGATCAATAGAAAATGAAGCAAGGCATTAACAAAAGGACACATACCAGGTTCAAGATAAATTTACCTGAAAGCCTGGAACGCGACCTCCCTGCTCTTCCTTCTTTGCCGGTAAGTTTTCTCTCCTCAAAAGCTTCACTCCCAGATCCTCCTCCTTGTCTGTCCCCGAAAAATGCCCCTTCTCTCCTGACCCACTTGTCTTTCTCAGCAGCCTCTCCTTCCTTTTTGTAATCAGACTGCGACCACCAGAAACCTCCAGCAACTAACTCCCCTCTTCCTCCTCCCGACCTCTTTTTTTTTTTTTTTTTTCATCCCACGTTCTTCTTCCTCTGTTTTTCCTTTAGCTTCTTTTTCTTTGGCTTCTCCCGCAAGTTTCTGCTGCCTCTCCCATCAGCCGCACTTCTCTGCCCGAGCCAACCTCCCATCAGCCGCCTGAAAAAACCAAGAAACTCCCCCTGCTTCGCAGCCTCAGGGGGGTCTACACGAGGCTTACAATTTTTAAAACATCAGAGAGAAACTTTTTTGGGTAGAGCCTTGAGTGGTTCCAACTTCCTCATCACCATGGCCAAATTCTCTCTCATGTCCATGGTCTCAAGAGTGATACTGCTGTCAAGTTGCCAGTCAAAATGGTAAACCAAGGACCCCAGAACAAGGTGCACCATCCGATGAGCTAATGGAACCCCGCACACATCCTTCGACCGGCTCCAAAGGATCAGCTCGAAATGTTGACCCTTATAATCAATATGGTTTAAGTCTAAAAACCTCTCAGGCTTGAAGGACGAAGGCTCCTCCCACAACTCCGCTTCCCTTCCGATGCTAAAACATTCACAAAAGCTGTGTGTTTTTGGAATATGGTATCCCATGAAATTGGTGTCTCGAACTGCCGTTAGGAACCAGAAATGGGATTGGAGGGTGCAACCTGAATGTTTCCTTCACGACAGCCTGTAAATATTGGAGATCATCAATGTGATTCTCTTCTAACTTACCGTTTGCTCCAACAACTCGACCGCCAAGCTCAGCTTTAACCTTCGCCATGCATTCGG

**Chr17 ancestral haplotype at breakpoint-1:**

***> VP11 gDNA + B_chr17_1F (17) + B_chr17_1R (18) primers***

***Sanger sequence color code (according to original positions in the TT genome assembly):***

**B_chr17: 5,100,855 – 5,101,772**

TTGATATTTGCCCACTTGGCCTGTTAATTTTTTTTCTTCAAAATTTTCAAATTTTATATATTTTTTCTCTTTCCAATTTTTCTCCTTTGATTTTTTTTTTCACTTTGCAATAACCAAATTATTTTGACCAACTAAAAATATTGTGACTTATTGGTCAAAGGAGAAAGTGAGAATATGAAATCCATTCTATCTTCTATTTATTATAGCATATCAATTAAATAAAATAAAATAATAACAAGCACAAAAAAAAGGTTGTACATGAGCTTATATTATGATGGGGGGAGAGCAACATTTAAAAAAAAGAAGTGATAACAGAGGAAGAAAATAACAAAGGTATTGTGGACCCCGCATTTTCGATCGCTCGAATGCGTTTCCCACTCGAATGGCGAAACTCGATTTTTATTTAAAAAAAATTGATTTATATTGATTATGAAAATGACTTGGAGTCGCCACTTATTTTTGTTTTATTTTTAAAAGGGTAAACAAAATAAGAAAGAAAAACCCTAAGTGTGACTCCTTGTTTTGGAAAAGGTGGTCTGTGAAAAACCGGATTGGGTTCGGGGGTCAGGTTACTTATCGGGAAGGTACGGTAGAGACCGTAGCACCCCTCTAAGTCCCTAAAGACAGGTCTCTACTAATAAAATGAAGCAATCATGGCAATTGATGAGGAAAACAATGAATACCTGGAACTATCATGCACATAGGAGAATCAAAACATGTATATGGAAATACCGGAATGGGAATAGATACGTACCTGGGTGACGAGCCACAATGCGCTTATCAAAAAGTGAGGTTAGTGCACAATTAAAGAATGATTTCAAGCATGTCGTAGGGCAGAATATAAACAATCATGCATGACAATTAAATCAAACAAACAATCAATCAATCATAAGGAAATCACATATGTGGGCCCCCAC

**Chr17 ancestral haplotype at breakpoint-2:**

***> VP11 gDNA + B_chr17_2F (19) + B_chr17_2R (20) primers***

***Sanger sequence color code (according to original positions in the TT genome assembly):***

**B_chr17: 5,123,530 – 5,124,692**

AAAGGACACATACCAGGTTCAAGATAAATTTACCTGAAAGTTCGGAACGCGACCTCCTCTGCTCTTCCTTCTTTGCCGGTAAGTTTTCTCTCCCCCTCAAAAGCTTCACTCCCAGATCCTCCTCCTTGTCTGTCCCCGAAAAAATGCCCTTCTCTCCTGACCCACTCCCTTGTCTTTCTCAGCAGCCTCTCCTTCCTTTTTGTAATCAGACTGCGACCACCAGAAACCTCCAGCAACTAACTCCCCTCTTCCTCCTCCCGACCTCTTTTTTTTTTTTTTTTTCCCCCCCCGTCCTCCTCCCCCGTTTTTCCTTTTACTTTTTTTTTTTTGGGTTCCCCCCGAAACCCCCTCGGCGCCCCCCCCCTTCGCCCGCTTTTTTTGCCCGAACCCACCCCCCCACCGCCCCCGAAAAAAAAACAAAAAAACCCCCCCCGGTTCCGCCCCCCCGGGGGGGCCCAACGGGAATATTTTTTTTTTTTTTGGGAACGGGGCCCCCCCCCCCCAACCCCTTTTTCTTTTATTAAAAAAAAAAAAAAAAAAAAGGGGAGAAAACAGGCATTTTGAGGAAATTTTCTTATTTCCCCTTTGGGCCGGAAACTTTTTTTTTCAAAATTTTCAAATTTTAATATTTTTTTTTCTCCGCCCGAAAATTTTTCCCCCTTTATTTTTTTTTTTTGTTTTTTATTTTCAAAAAACCCAATTTTTTTTACCAAAAAAAAATATTTTGGCTCTTTATTGCTCAAGAAGGGGAGGGAAGAATAGAACACCCCTTCCTTTTTTTTTTTTTTTAGGGCACCCCTTTTTAAAAAAAAAAAAAAAAACCAACCCAAAAAAAAAAATTGGGCACAACACTTTATTATATAGTGGGGGCGTCACCCATTTTTAGAAAAAGGGGGGGGAAAAAAAGAAAAAAAAAAAAGGGTATATATTTTTGGTTTTTTTGGGAAAGGGCCCCCCCCCCCCAATCTTTTTTTCTTTTTTTTAAAAAAAAAAAAAAAAAAAGAGGGGAGCACCAACTTAAAAGAAATTTTTTTTTTCCCCCCCGGGGCGGAAATTTTTTCTCCAAAAATCACAATTTTTAAATTTTTTTCCCCCCCAATTTTCCCCCTGGAGTTTTTTTTTCCTCTTTGGAAACCACAAATTATTTCCCCACATAAAAAAT

**Chr08 ancestral haplotype:**

***> VP11 gDNA +*** ***A_chr08_1F (15) + A_chr08_2R (16) primers***

***Sanger sequence color code (according to original positions in the TT genome assembly):***

**A_chr08: 20,029,691 – 20,030,607**

GTGCTTGATCTATTTATGAATCCAATAATAAAAATCAATTTATCATGTTAGGAGTAGACCAATAACAACTTGTCATCTTCTATATGAAAAAGGTTAATTTTAAATGAAAAATAATTAAAATTTTAGAGTAGATATCATGAAACAGAGACATAAATCAATAATAACCGTTCCAAAAATTAAGAGCACTTTTTAATACAATGGAGAGGAATCTTTAGCATTCCGTATAATATATCTATTTTTGTGATTATTGCAACAAATTAAAATAAACTCAAATTATTCCATAAATATATTAATTCCTCAAATAAACATACAGTAGTTTATTTCTACGTACTTTTGAGTGATGAGGCTTACAATTTTTAAAACATCAGAGAGAAACTTTTTTGGGTAGAGCCTTGAGTGGTTCCAACTTCCTCATCACCATGGCCAAATTCTCTCTCATGTCCATGGTCTCAAGAGTGATACTGCTGTCAAGTTGCCAGTCAAAATGGTAAACCAAGGACCCCAGAACAAGGTGCACCATCCGATGAGCTAATGGAACTCCCGCACACATCCTTCGACCGGCTCCAAAGGGGATCAGCTCGAAATGTTGACCCTTATAATCAATATGGTTTAAGTCTAAAAACCTCTCAGGCTTGAAGGACGAAGGCTCCTCCCACAACTCCGCTTCCCTTCCGATGGCCCAAACATTCACAAAAAGCTGTGTGTTTTTGGGAATATGGTATCCCATGAAATTGGTGTCTCGAACTGCCTTCCGAGGAACCAGAAATGGGATTGGAGGGTGCAACCTGAATGTTTCCTTCACGACAGCCTGTAAATATTGGAGATCATCAATGTGATTCTCTTCTAACTTACCGTTTGCTCCAACAACTCGACCAAGCTCAGCTTTAACCTTCGCCATGCATTCGGGATGCGAAAG
