# Supplementary material for: A complex reciprocal translocation is linked to reduced gamete viability in a loose-bunch grapevine somatic variant
Source: BMC Plant Biol. 2026 Jan 27;26:348. doi: 10.1186/s12870-026-08212-7 (PMC12918644; doi:10.1186/s12870-026-08212-7)
Supplement: Supplementary file 3 — Supplementary Material 3. Supplementary Figure S1. Summary of ONT whole-genome sequencing reads produced for VP11 and RJ51 Tempranillo clones. Supplementary Figure S2. IGV visualization of ONT read alignments supporting Dup11to7 and Dup17to8 interspersed duplications specific to VP11 clone. Supplementary Figure S3. Uncropped images of the DNA electrophoresis gels used for molecular validation of structural variants. Supplementary Figure S4. Validation of Tra1-3 phasing by genotyping a 5.8 kb insertion polymorphism at the Tra1-3 breakpoint region in chromosome 3. Supplementary Figure S5. Design of PCR-based analysis of co-segregation between chromosome 1 and 3 proximal and distal segments resulting from Tra1-3 translocation. Supplementary Figure S6. Co segregation analysis between the presence or absence of VP11 structural variation events and reproductive-related phenotypes in VP11 self-cross progeny. Supplementary Figure S7. Effect of flower sex type on bunch compactness in VP11 and RJ51 self-cross populations. [file 12870_2026_8212_MOESM3_ESM.pdf]

SUPPLEMENTARY FIGURES

A complex reciprocal translocation is linked to reduced gamete viability in a loose-bunch grapevine somatic variant

Noelia Alañón-Sánchez[1], Yolanda Ferradás[1,2], Ilja Bezrukov[3], Detlef Weigel[3,4], Pablo Carbonell-Bejerano[1*], Javier Ibáñez[1*]

[1] Instituto de Ciencias de la Vid y del Vino (ICVV; CSIC, Gobierno de La Rioja, Universidad de La Rioja), 26007 Logroño, Spain

[2] Current address: Facultade de Bioloxía, Universidade de Santiago de Compostela, 15872 Santiago de Compostela, Spain

[3] Max Planck Institute for Biology Tübingen, 72076 Tübingen, Germany

[4] Institute for Bioinformatics and Medical Informatics, University of Tübingen, 72076 Tübingen, Germany

* Corresponding authors: pablo.carbonell@icvv.es, javier.ibanez@icvv.es

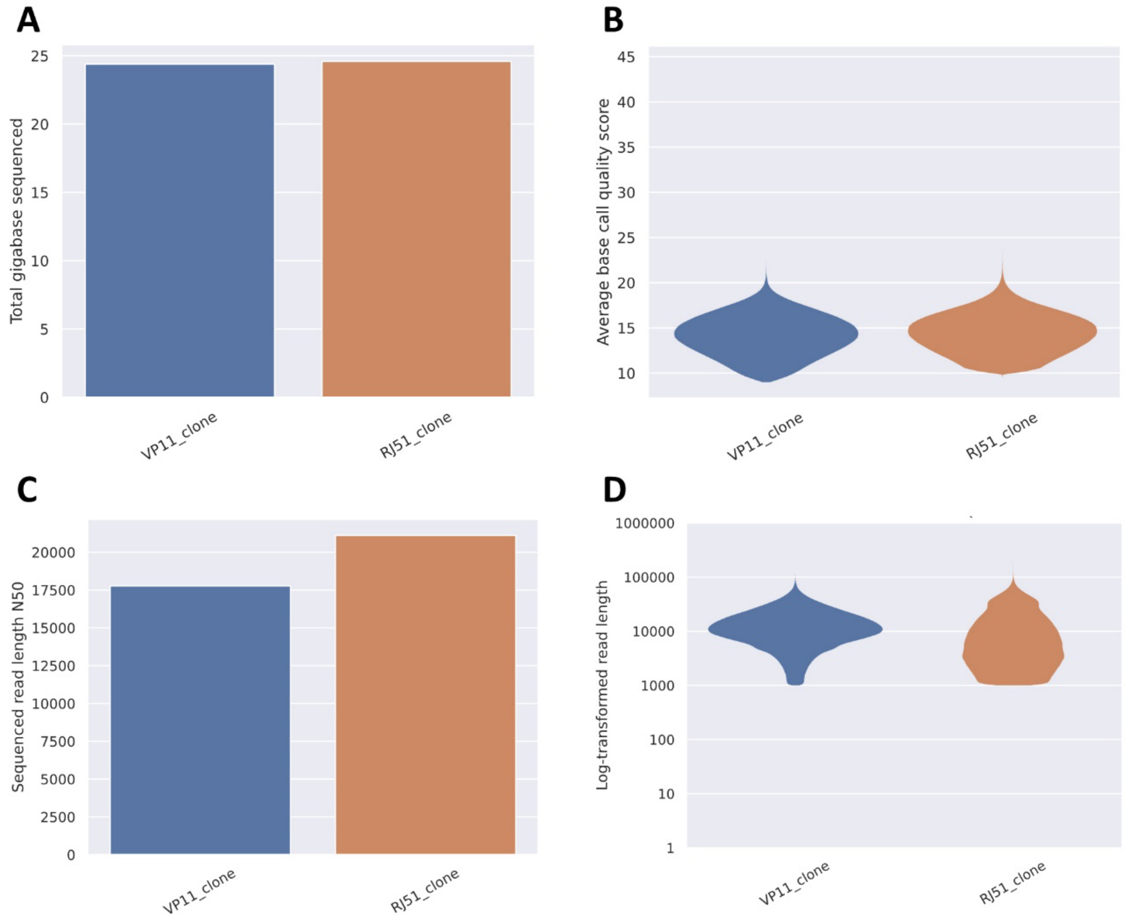

Supplementary Figure S1. Summary of ONT whole-genome sequencing reads produced for VP11 and RJ51 Tempranillo clones. Summary metrics for the filtered reads (Length >1kb and Q >9) used for structural variation calling. A) Total sequence (Gb). Read base call quality distribution (qv). C) Read length N50 (bp). D) Read length distribution (bp).

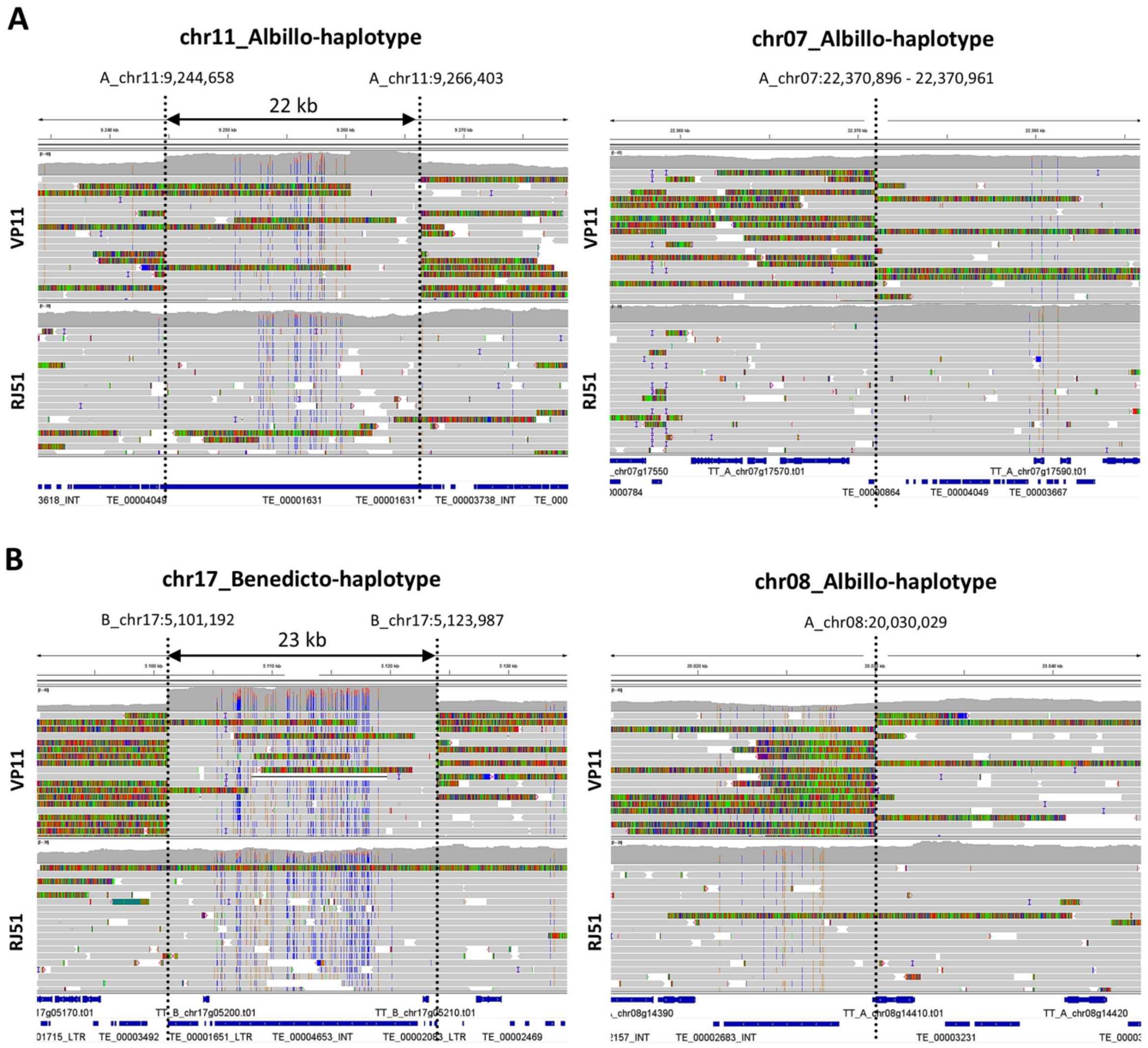

Supplementary Figure S2. IGV visualization of ONT read alignments supporting Dup11to7 and Dup17to8 interspersed duplications specific to VP11 clone. Reads from VP11 and RJ51 clones aligned to the diploid genome assembly of TT cultivar are shown. A) Read alignments at Dup11to7 breakpoints. B) Read alignments at Dup17to8 breakpoints. ONT reads with soft-clipping alignment (denoted in colors different than grey) specific of VP11 clone support the position of the breakpoints. Duplications in both Dup11to7 and Dup17to8 are supported by increased read coverage in VP11 but not in RJ51, as shown in the upper IGV coverage panel. Annotated genes and TEs are shown as blue rectangles in the lower section of the figure.

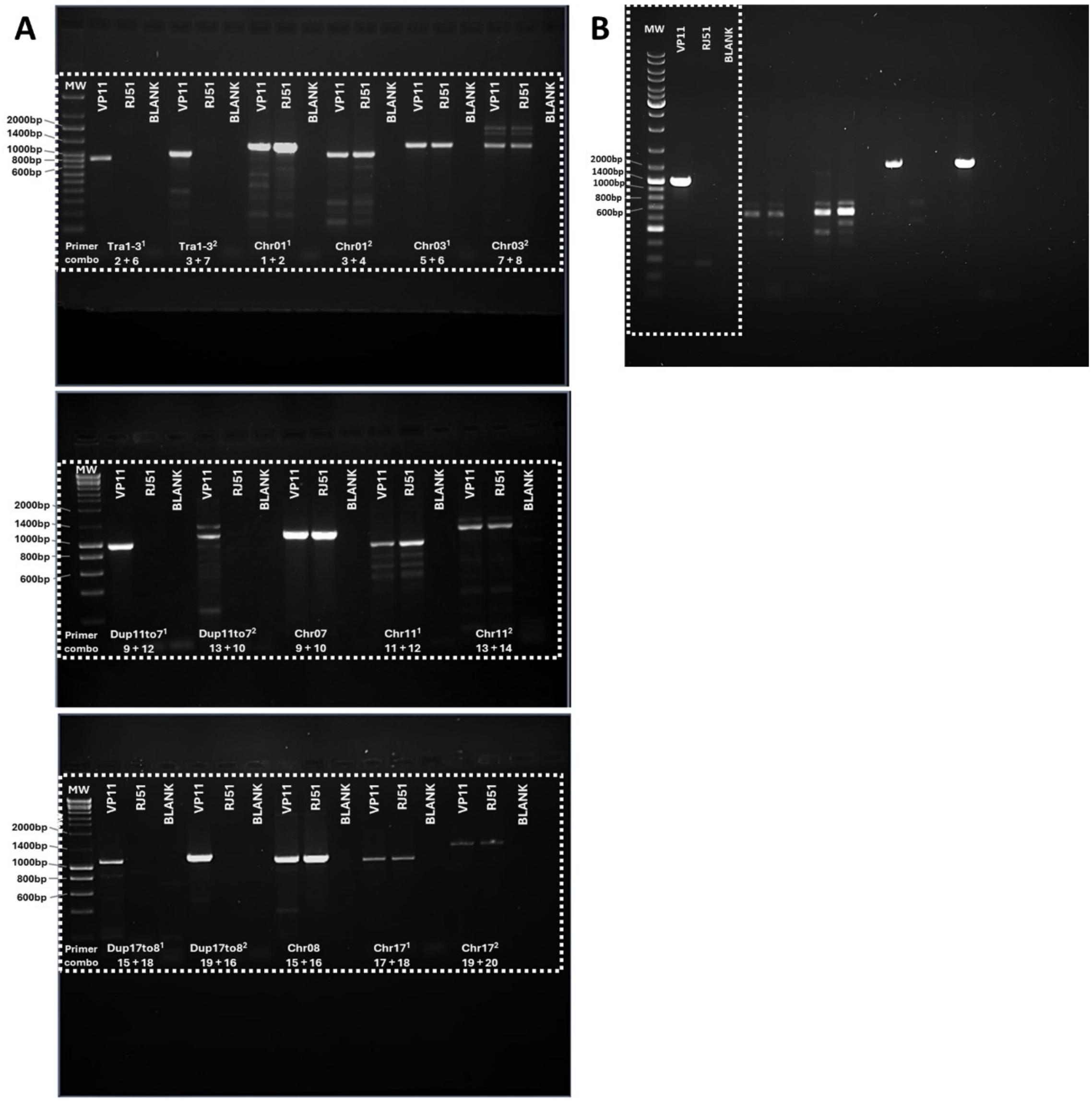

Supplementary Figure S3. Uncropped images of the DNA electrophoresis gels used for molecular validation of structural variants. Dashed lines indicate the cropping was carried out to retrieve the images used in Figures 3 and S3. A) Original images of three electrophoresis gels used for SV validation in Figure 3. PCR amplicons generated using primers designed to validate the presence of SVs and the corresponding ancestral chromosomes (without SVs) in VP11 and RJ51 clones. B) Original image of the electrophoresis used for SV phasing validation in Figure S4. PCR amplicons generated with primers designed on the 'Albillo Mayor' haplotype, tested in VP11 and RJ51. Extra lanes to the right correspond to additional primer pairs tested that either confirmed the result or were discarded as unspecific because several unexpected bands were amplified in both clones.

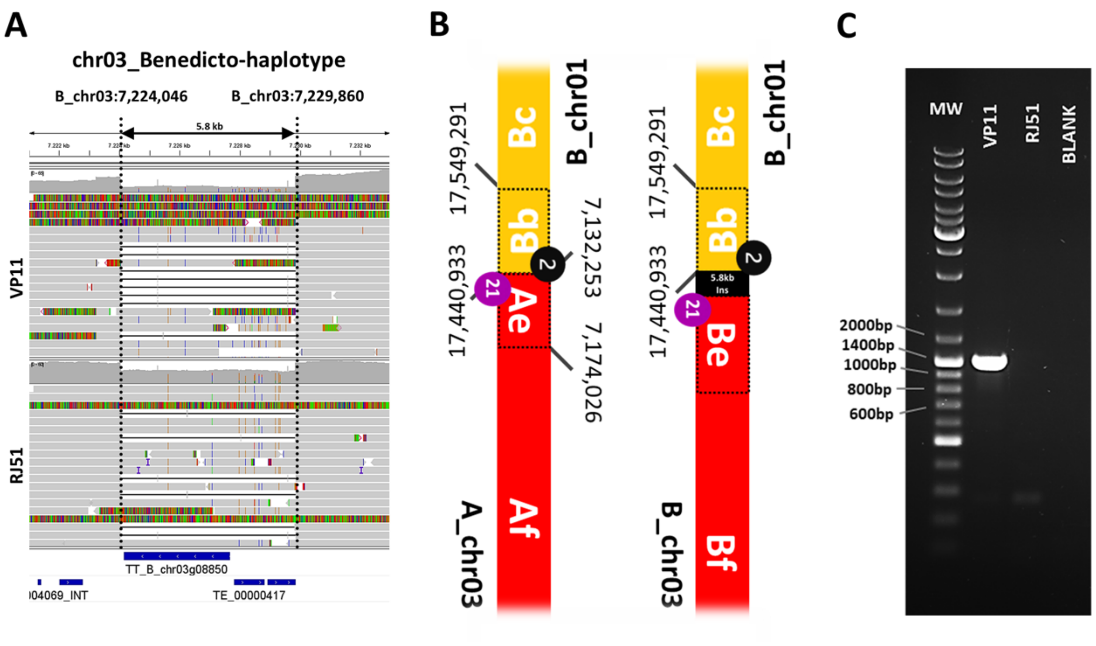

Supplementary Figure S4. Validation of Tra1-3 phasing by genotyping a 5.8 kb insertion polymorphism at the Tra1-3 breakpoint region in chromosome 3. A) IGV capture showing ONT read alignments to the diploid genome assembly of TT in the 'Benedicto' haplotype of chromosome 3. The 5.8 kb insertion is labelled with a black line delimiting splitting reads of the 'Albillo Mayor' haplotype aligned in this region. B) Schematic representation of the two phasing alternatives of Tra1-3, highlighting the Tra1-3 breakpoints and the 5.8 kb InDel affecting this locus in TT (black box shown in B_chr03). Encircled numbers represent PCR primer positions. C) Agarose gel electrophoresis of PCR amplicons generated with primers designed on the 'Albillo Mayor' haplotype, tested in clones VP11 and RJ51. Blank lane corresponds to the PCR negative control (without template). The uncropped image of the electrophoresis gel shown in Fig. S3C is presented in Fig. S2B.

A

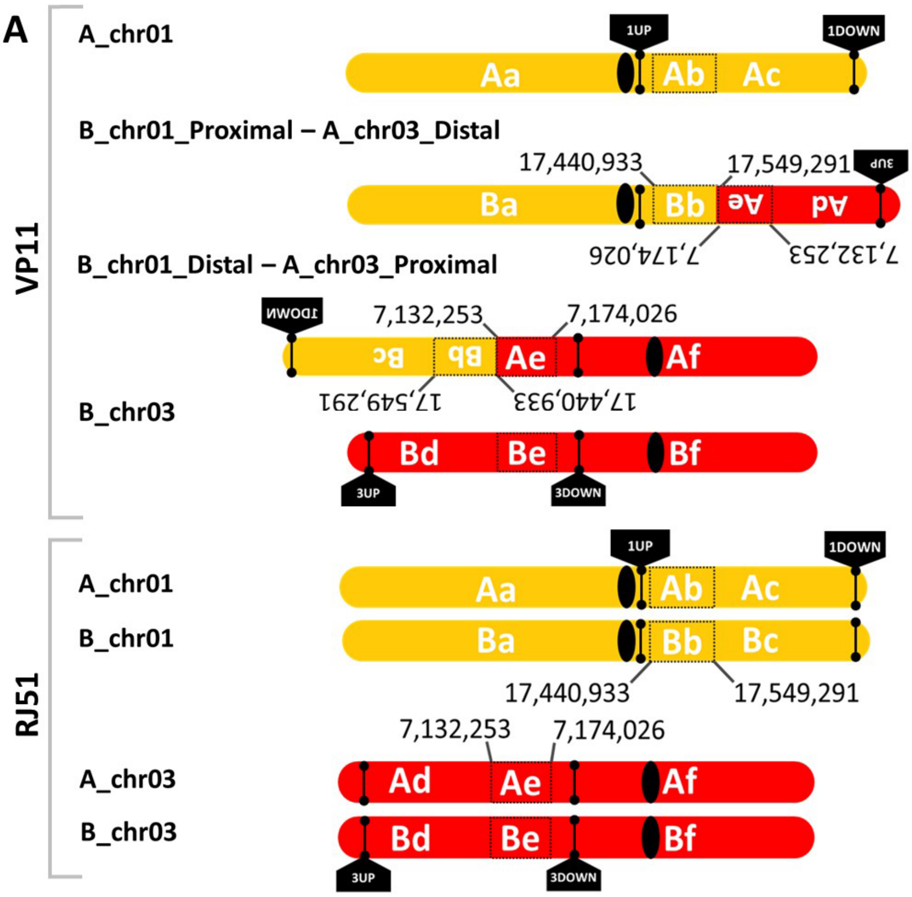

B

PRIMER NAME	LOCATION	
1UP_F	B_chr01:16,211,341-16,211,364 (+)	A_chr01:17,408,505-17,408,528 (+)
1UP_R	B_chr01:16,212,424-16,212,448 (-)	A_chr01:17,409,600-17,409,624 (-)
1DOWN_F	B_chr01:23,748,709-23,748,728 (+)	A_chr01:25,160,613-25,160,632 (+)
1DOWN_R	B_chr01:23,749,746-23,749,766 (-)	A_chr01:25,161,650-25,161,670 (-)
3UP_F	A_chr03:2,066,755-2,066,774 (+)	B_chr03:2,001,972-2,001,991 (+)
3UP_R	A_chr03:2,067,878-2,067,899 (-)	B_chr03:2,003,086-2,003,107 (-)
3DOWN_F	A_chr03:8,076,065-8,076,084 (+)	B_chr03:8,036,835-8,036,854 (+)
3DOWN_R	A_chr03:8,082,229-8,082,248 (-)	B_chr03:8,045,191-8,045,210 (-)

Supplementary Figure S5. Design of PCR-based analysis of co-segregation between chromosome 1 and 3 proximal and distal segments resulting from Tra1-3 translocation. Primer pairs were designed to amplify SNP-rich regions to enable identification of inherited haplotypes in the S_1 progeny of VP11 and RJ51 clones. A) Diagram of chromosomes 1 and 3 from the 'Albillo Mayor' and 'Benedicto' haplotypes in clones VP11 and RJ51, and position of 1UP, 1DOWN, 3UP, and 3DOWN SNP-rich amplicons. The number in the amplicon names denote the ancestral chromosome where the SNP-rich amplicon locates, while UP and DONW indicate its relative position to the Tra1-3 breakpoint. B) The coordinates of the SNP-rich amplicon PCR primers on each haplotype of the TT genome assembly are indicated.

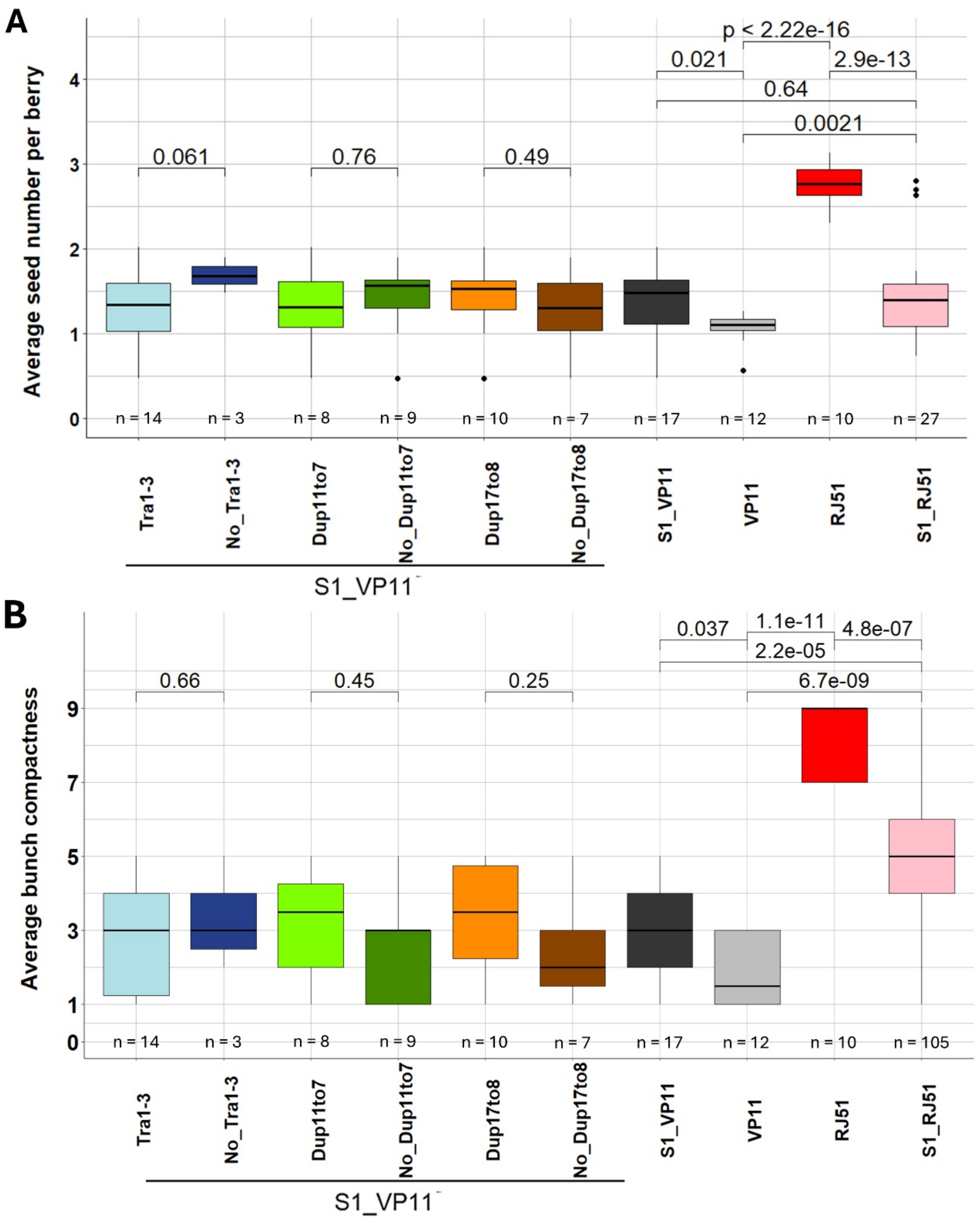

Supplementary Figure S6. Co-segregation analysis between the presence or absence of VP11 structural variation events and reproductive-related phenotypes in VP11 self-cross progeny. A) Analysis of seed number per berry. B) Analysis of bunch compactness. Bunches were collected at maturity over two consecutive years. Ten berries per bunch were collected from to estimate seed number. In box plots, the horizontal black line represents the median value, the colored

interval is the interquartile range showing the middle 50% of data, and the bars represent lower and upper quartiles. T-tests were used to determine statistical differences between samples.

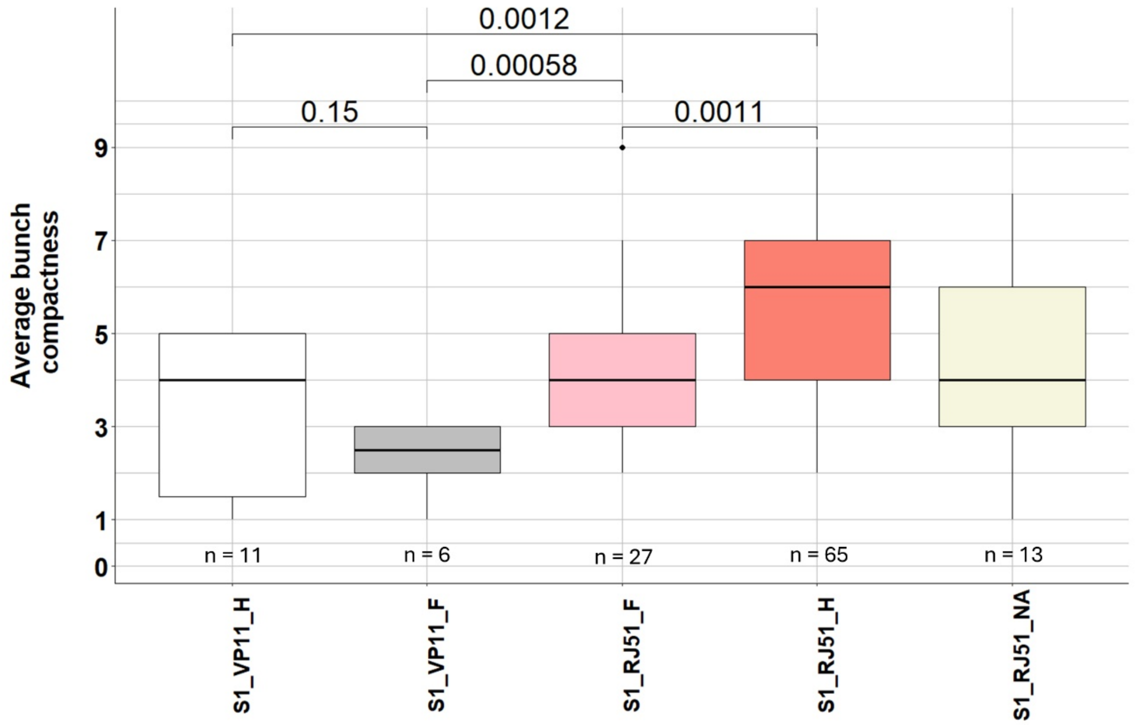

Supplementary Figure S7. Effect of flower sex type on bunch compactness in VP11 and RJ51 self-cross populations. For each population, female and hermaphrodite individuals were compared. S1_VP11_F, VP11 S_1 individuals with female flowers; S1_VP11_H, VP11 S_1 individuals with hermaphrodite flowers; S1_RJ51_F, RJ51 S_1 individuals with female flowers; S1_RJ51_H, RJ51 S_1 individuals with hermaphrodite flowers and S1_RJ51_NA, RJ51 S_1 individuals on which flower sex was not identified. Bunches were collected at maturity over two consecutive years. In box plots, the horizontal black line represents the median value, the colored interval is the interquartile range showing the middle 50% of data, and the bars represent lower and upper quartiles. T-tests were used to determine statistical differences between samples.
